# Dynamic monitoring of cerebrospinal fluid circulating tumor DNA to identify unique genetic profiles of brain metastatic tumors and better predict intracranial tumor responses in non-small cell lung cancer patients with brain metastases: a prospective cohort study (GASTO 1028)

**DOI:** 10.1186/s12916-022-02595-8

**Published:** 2022-11-14

**Authors:** Meichen Li, Jing Chen, Baishen Zhang, Juan Yu, Na Wang, Delan Li, Yang Shao, Dongqin Zhu, Chuqiao Liang, Yutong Ma, Qiuxiang Ou, Xue Hou, Likun Chen

**Affiliations:** 1 Department of Medical OncologyState Key Laboratory of Oncology in South ChinaCollaborative Innovation Center for Cancer Medicine, Sun Yat-Sen University Cancer Center, Guangzhou, China; 2 Department of Oncology, The First Affiliated Hospital of Jinan University, Guangzhou, China; 3 Chemotherapy Department 2, Zhongshan City People’s Hospital, Zhongshan, China; 4 School of Public Health, Nanjing Medical University, Nanjing, China; 5 Nanjing Geneseeq Technology Inc, Nanjing, China

**Keywords:** Cerebrospinal fluid, Circulating tumor DNA, Brain metastases, Intracranial response, Clonal evolution

## Abstract

**Background:**

Due to the blood-brain barrier, plasma is not an ideal source to evaluate the genetic characteristics of central nervous system tumors. Thus, cerebrospinal fluid (CSF) is becoming an alternative biopsy type to evaluate the genetic landscape of intracranial tumors. We aimed to explore the genetic profiles of CSF-derived circulating tumor DNA (ctDNA) to predict intracranial tumor responses and monitor mutational evolution during the treatment of non-small cell lung cancer (NSCLC) patients with brain metastases.

**Methods:**

We conducted a prospective study of 92 newly diagnosed NSCLC patients with brain metastases. Paired CSF and plasma samples were collected at baseline, 8 weeks after treatment initiation, and disease progression. All samples underwent next-generation sequencing of 425 cancer-related genes.

**Results:**

At baseline, the positive detection rates of ctDNA in CSF, plasma, and extracranial tumors were 63.7% (58/91), 91.1% (82/90), and 100% (58/58), respectively. A high level of genetic heterogeneity was observed between paired CSF and plasma, while concordance in driver mutations was also observed. A higher number of unique copy number variations was detected in CSF-ctDNA than in plasma. ctDNA positivity of CSF samples at baseline was associated with poor outcomes (HR=2.565, *P*=0.003). Moreover, patients with ≥ 50% reductions in the concentrations of CSF ctDNA after 8 weeks of treatment had significantly longer intracranial progression-free survivals (PFS) than patients with < 50% reductions in CSF ctDNA concentrations (13.27 months vs 6.13 months, HR=0.308, *P*=0.017). A ≥ 50% reduction in CSF ctDNA concentrations had better concordance with radiographic intracranial tumor responses than plasma. A ≥ 50% reduction in plasma ctDNA concentrations was also associated with longer extracranial PFS (11.57 months vs 6.20 months, HR=0.406, *P*=0.033). Based on clonal evolution analyses, the accumulation of subclonal mutations in CSF ctDNA was observed after 8 weeks of treatment. The clonal mutations that remained in more than 80% in CSF after 8 weeks also predicted shorter intracranial PFS (HR=3.785, *P*=0.039).

**Conclusions:**

CSF ctDNA exhibited unique genetic profiles of brain metastases, and dynamic changes in CSF ctDNA could better predict intracranial tumor responses and track clonal evolution during treatment in NSCLC patients with brain metastases.

**Trial registration:**

ClinicalTrials.gov identifier: NCT 03257735.

**Supplementary Information:**

The online version contains supplementary material available at 10.1186/s12916-022-02595-8.

## Background

Brain metastases occur in nearly 30–40% of lung cancer patients and always result in poor clinical outcomes [1]. The treatment strategies for lung cancer patients have become increasingly precise due to evaluations of molecular tumor characteristics. Thus, identifying actionable alterations and choosing optimal treatments have become increasingly important [2]. Accumulating evidence has revealed the genetic heterogeneity and different clinical responses between primary lung tumors and metastatic brain tumors [3–5]. As obtaining intracranial tumor samples is invasive and risky, there is an urgent need to identify alternative biopsy types to evaluate the genetic landscape and tumor evolution of central nervous system (CNS) tumors.

Circulating tumor DNA (ctDNA) is a promising biomarker to characterize the mutational profiles of tumors. Through real-time dynamic monitoring, ctDNA can also be used to trace tumor evolution and treatment responses, and identify resistance mechanisms [5, 6]. However, due to the blood-brain barrier, plasma is not an ideal source to evaluate the genetic characteristics of CNS tumors [7]. Thus, cerebrospinal fluid (CSF), which can be collected through a minimally invasive lumbar puncture procedure, is becoming an appealing surrogate as it is contained throughout the CNS and is in contact with intracranial lesions [8]. Recent studies have shown that CSF-ctDNA represents the genetic alterations of brain tumors better than plasma, and also represents the changes in brain tumor burden [9]. However, most current studies included very few samples and were retrospectively designed. No study has reported the use of CSF ctDNA to predict intracranial responses or to monitor mutational evolution during treatment.

We conducted this prospective cohort study to compare the genetic landscapes of paired CSF and plasma, and sought to trace intracranial tumor evolution and treatment responses through serial ctDNA sequencing of paired CSF and plasma samples in non-small cell lung cancer (NSCLC) patients with brain metastases.

## Methods

### Study design and participants

This prospective study (ClinicalTrials.gov identifier: NCT 03257735) was designed and performed at the Sun Yat-sen University Cancer Center (Guangzhou, China). Main inclusion criteria included following: (1) NSCLC newly diagnosed by histopathology; (2) brain metastases confirmed by enhanced brain MRI at primary diagnosis, with at least one intracranial lesion whose longest diameter was > 5 mm; (3) treatment-naïve (no previous systemic therapy, surgery, or radiotherapy); (4) brain metastases were asymptomatic or responding to corticosteroid treatment, followed by systemic therapy as the first-line treatment; and (5) had no contraindication for lumbar puncture. All patients meeting the inclusion criteria were enrolled consecutively between January 2017 and December 2020 at our institution. For first-line treatment, patients received systemic therapies based on their mutational characteristics. Paired CSF and blood samples with or without primary extracranial tumor samples were collected at baseline (before treatment), 8 weeks after the initiation of first-line treatment, and at the time of disease progression. At each time point, 5 ml of CSF and 8 ml of blood were collected, and radiographic evaluations were performed within one week (i.e., enhanced computerized tomography scans for extracranial lesions and enhanced magnetic resonance imaging for intracranial lesions). Radiographic evaluations were then performed at approximately 8-week intervals until tumor progression. The clinical response was assessed according to the Response Evaluation Criteria in Solid Tumors (RECIST), version 1.1 by a radiologist who was blinded to the patients’ clinical information. This study was approved by the ethics committee of the Guangdong Association Study of Thoracic Oncology (GASTO ID:1028, Approval No. A2017-003). All patients provided written informed consent to participate in the study and provide samples for tumor genetic profiling.

### Next-generation sequencing and data processing

CSF and whole blood samples were collected in cell-free DNA BCT tubes (Streck Inc., La Vista, NE, USA). Within 24 h of CSF and blood collection, the cellular fraction was removed by two-step centrifugation at 4°C (1900 g for 10 min within 2 h of collection and 16,000 g for 10 min). The white blood cells were used for genomic DNA extraction (DNeasy Blood & Tissue Kit, Qiagen) as the germline controls. Samples were stored at -80°C until further processing. CtDNA was extracted using the QIAamp Circulating Nucleic Acid Kit (Qiagen), as previously reported [10], and analyzed using comprehensive genomic profiling of 425 cancer-related genes in a central testing laboratory (Nanjing Geneseeq Technology, Jiangsu, China), as previously described [10–13]. For patients with extracranial tumor tissues, genomic DNA was also extracted from tissue biopsy samples using the QIAamp DNA FFPE Tissue Kit (Qiagen) and subjected to next-generation sequencing with the same panel described above. Sequencing was performed on the Illumina HiSeq4000 platform and data analysis was performed as previously described [10–12]. In brief, sequencing data were analyzed by Trimmomatic [14] to remove low-quality (quality < 15) or N bases, and then mapped to the human reference genome hg19 using the Burrows-Wheeler Aligner (https://github.com/lh3/bwa/tree/master/bwakit). PCR duplicates were removed by Picard (available at: https://broadinstitute.github.io/picard/). The Genome Analysis Toolkit (GATK) (https://software.broadinstitute.org/gatk/) was used to perform local realignments around indels and base quality reassurance. Single nucleotide polymorphisms (SNPs) and indels were analyzed by VarScan2 [15] and Haplotype Caller/Uni edGenotyper in GATK, with the mutant allele frequency (MAF) cutoff of 0.2% for cfDNA samples, and a minimum of three unique mutant reads. Common SNPs were excluded if they were present in > 1% population frequency in the 1000 Genomes Project or the Exome Aggregation Consortium (ExAC) 65,000 exomes database. The resulting mutation list was further filtered by an in-house list of recurrent artifacts based on a normal pool of whole blood samples. Gene fusions were identified by FACTERA [10, 16–18]. The medium depth of coverage after the removal of PCR duplicates was > 2000× and > 500× for liquid biopsies and tissue samples, respectively.

The ctDNA levels were calculated as previously described [19, 20]: maxVAF = max variant allele frequency; ctDNA concentration (hGE/mL) = mean ctDNA VAF * cell-free DNA concentration (pg/mL) / 3.3, which assumes that each haploid genomic equivalent (hGE) weighed 3.3 pg.

### Analysis of the consistency of genetic mutations

To evaluate the mutations that were consistently identified in the paired samples (sample A and sample B), the overlapping genes and unique genes from each sample were illustrated using a Venn diagram. Consistency was calculated using the following equation:

$$\mathrm{Consistency of sample A to sample B }\left(\%\right) = \frac{A \cap B}{A}\times 100\%$$

Consistency was analyzed for all mutant-positive samples. When considering the factors of the somatic mutations and driver gene variants, consistency was analyzed after removing the copy number variants (CNVs) or non-driver gene variants from the samples.

### Mutation clonality analysis

At baseline, a mutation was considered clonal if its VAF was more than 25% of the maxVAF, and it was defined as subclonal if it was below this threshold [21]. Newly acquired mutations in post-treatment samples were always defined as subclonal mutations.

### Statistical analysis

Survival data were analyzed using Kaplan-Meier curves and Cox proportional hazard regression analyses. Comparisons of the continuous variables were made using an unpaired two-tailed t-test, while comparisons of the categorical variables between groups were performed using the chi-squared test or Fisher’s exact test. The concordance of genomic alterations between CSF and plasma, and concordance of dynamic changes in ctDNA and radiographic response was assessed using Cohen’s kappa coefficient. All statistical analyses were performed using R software (version 3.5.3). Two-tailed *p*-values of less than 0.05 were considered statistically significant.

## Results

### Baseline characteristics of the study cohort

**Fig. 1:**
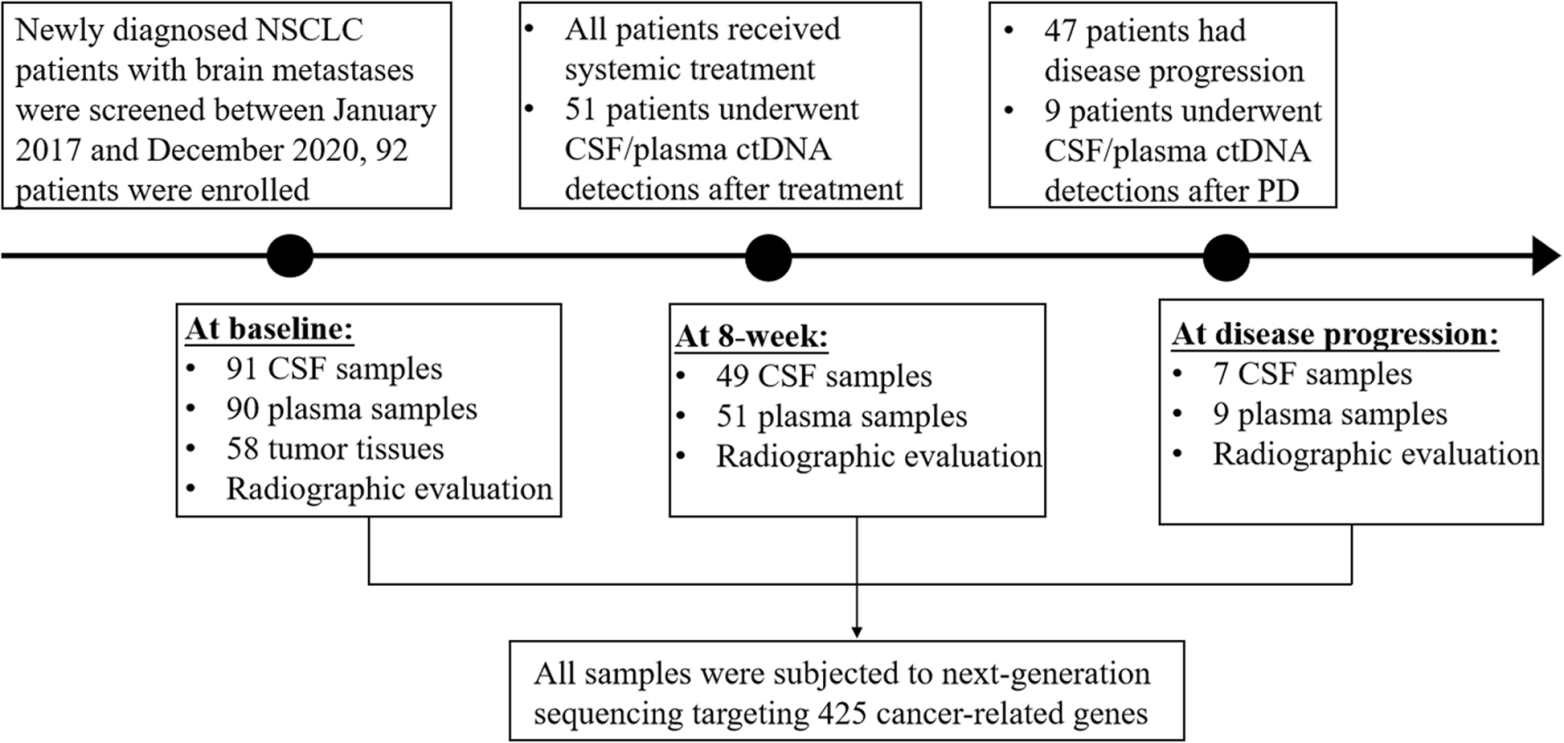
Flowchart of the study design and participants. The study design and sample collection timepoints are shown with the number of patients and available samples. Abbreviations: NSCLC, non-small cell lung cancer; CSF, cerebrospinal fluid

The study flow chart is shown in Fig. 1. A total of 92 NSCLC patients with brain metastases were enrolled in the study. The baseline characteristics of patients are summarized in Table 1. The median age was 57 years (range, 28–74 years), 59 patients (64.1%) were male, and 78 patients (84.8%) had lung adenocarcinoma. All patients received systemic therapies as their first-line treatment, 33 patients (35.9%) received chemotherapy, 10 patients (10.8%) received immunotherapy, and 49 patients (53.3%) received tyrosine kinase inhibitors (TKIs).

**Table 1:** Clinical characteristics of the study cohort at baseline (*N* = 92).

**Characteristics**	**No. of patients (%)**
**Age**, median (range)	57 (28–74)
**Sex**	
Male	59 (64.1)
Female	33 (35.9)
**Histological type**	
Adenocarcinoma	78 (84.8)
Non-adenocarcinoma	14 (15.2)
**Smoking status**	
Smokers	41 (44.6)
Non-smokers	51 (55.4)
**ECOG PS**	
0–1	63 (68.5)
≥ 2	29 (31.5)
**CNS symptoms**	
Yes	49 (53.3)
No	43 (46.7)
**Brain tumor number**	
1–3	31 (33.7)
≥ 4	61 (66.3)
**Maximum brain tumor size (mm)**	
Median (range)	19.0 (5.0-47.4)
**Thoracic stage **^**a**^	
I–II	12 (13.0)
IIIA	29 (31.5)
IIIB	40 (43.5)
IIIC	11 (12.0)
**Distant metastases**	
Liver	11 (12.0)
Bone	45 (48.9)
Adrenal gland	19 (20.7)
**Gene mutation**	
EGFR mutation	44 (47.8)
ALK rearrangement	3 (3.3)
EGFR/ALK wild-type	45(48.9)
**Lung-molGPA **^**b**^	
0–1	8 (8.7)
1.5–2	23 (25.0)
2.5–3	44 (47.8)
3.5–4.5	17 (18.5)
**First-line treatment**	
Chemotherapy	35 (38.0)
Immunotherapy	10 (10.9)
Tyrosine kinase inhibitors (TKIs)	47 (51.1)
**With tumor tissues**	58 (63.0)
Primary lung tumors	38 (41.3)
Lymph nodes	20 (21.7)
**Without tumor tissues**	34 (37.0)

Abbreviations: *ECOG PS*, Eastern Cooperative Oncology Group performance status; *CNS*, central nervous system; *TKIs*, tyrosine kinase inhibitors

^a^Thoracic stage was calculated according to 8th American Joint Committee on Cancer (AJCC) staging system

^b^An update of the diagnosis-specific Graded Prognostic Assessment (DS-GPA) using molecular markers

In total, 91 CSF samples and 90 plasma samples were collected from 92 patients at baseline (before treatment), and 58 patients had paired extracranial tumor tissues at baseline (38 primary lung tumor tissues and 20 lymph node tissues). Then, 49 CSF samples and 51 plasma samples were collected from 51 patients 8 weeks after the initiation of first-line treatment. Seven CSF samples and nine plasma samples were collected from nine patients at disease progression (Fig. 1).

### Concordance of genomic alteration detection in baseline CSF, plasma, and tumor tissue samples

**Fig. 2:**
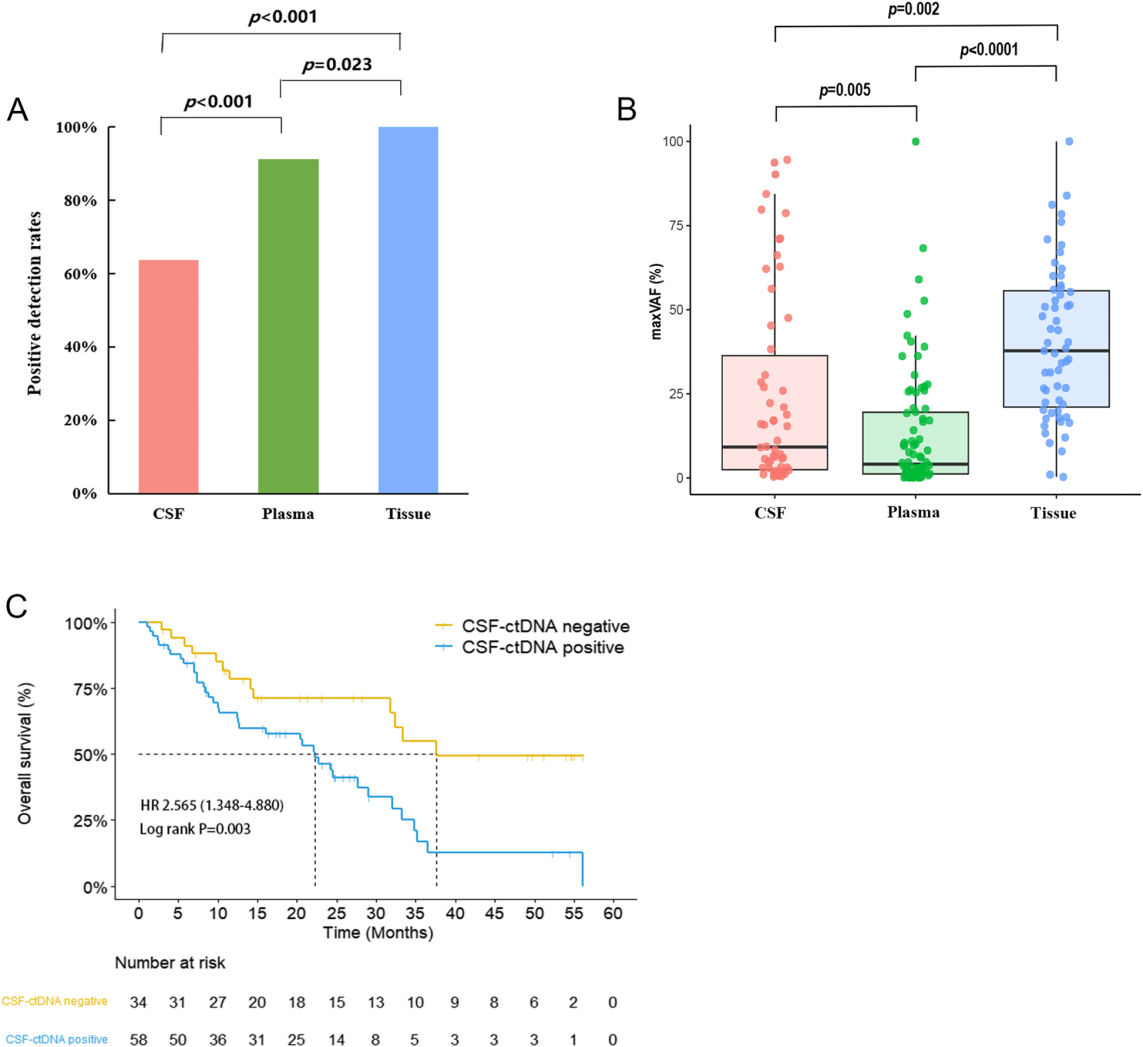
The detection of ctDNA at baseline and the correlation between overall survival and baseline CSF ctDNA positivity. **A** Positive detection rates of somatic mutations in CSF, plasma, and tissue samples. *P*-values were calculated using the Fisher’s exact test. **B** The distribution of max VAF of the three sample types. *P*-values were calculated using an unpaired two-tailed *t*-test. **C** Overall survival of patients with positive and negative CSF ctDNA detection at baseline. *P*-values were calculated using the log-rank test. Abbreviations: CSF, cerebrospinal fluid; ctDNA, circulating tumor DNA; HR, hazards ratio; VAF, variant allele frequency

At baseline, we detected at least one somatic mutation in 63.7% (58/91) of CSF samples, 91.1% (82/90) of plasma samples, and 100% (58/58) of extracranial tumor tissues (Fig. 2A). However, the max VAF in CSF ctDNA-positive samples and tumor tissues were significantly higher than that in plasma (Fig. 2B). A subgroup of 57 patients who had paired tissue-plasma-CSF samples at baseline were compared in three aspects (Additional file 1: Fig. S1A-C). All baseline tissue samples were positive for genomic alterations and 96.5% (55/57) of which carried driver/oncogenic mutations. Comparatively, CSF was inferior to tumor tissue for positive detection rate of any genomic alteration. Only 67% of the CSF samples were positive for genomic aberrations, 44% contained mutations in cancer driver genes, such as *EGFR*, *ALK*, *MET*, *BRAF*, *ROS1*, *NTRK1*, *RET*, *KRAS*, and *ERBB2 *[22], and 51% contained oncogenic mutations defined by the oncoKB database [23]. Over half (52%, 246/474) of the genomic alterations detected in tissue samples were unique, while the percentage of unique genomic alterations identified in CSF (24.5%, 40/163) and plasma (24.4%, 73/299) samples were comparable (Additional file 1: Fig. S1D). Genomic alterations shared by all three sample types accounted for 56% (91/163) of alterations detected in CSF, compared to 19% for tumor tissue (91/474). When considering only driver gene mutations (Additional file 1: Fig. S1E), the concordance rate for CSF was 78% versus tissue, and 91% for plasma versus tissue, which was comparable to oncogenic mutations (CSF vs. tissue: 85%; plasma vs. tissue: 84%; Additional file 1: Fig. S1F). We also investigated the consistency of genomic alterations, including somatic mutations and CNVs, between tumor tissues and liquid biopsies. An average of 79.7% (range: 0-100%) of genomic alterations from primary lung tumors and 70.7% (range: 0–100%) from lymph nodes were detected in paired plasma samples, respectively. However, only 59.7% (range: 0–100%) of genomic alterations from primary lung tumors and 52.3% (range: 0-100%) from lymph nodes were detected in paired CSF samples (Additional file 2: Fig. S2A). When only considering somatic mutations, a higher level of heterogeneity was observed between CSF and paired primary tumors after excluding CNVs (Additional file 2: Fig. S2B).

Patients with ctDNA detected in the baseline CSF samples had significantly shorter overall survivals than patients without ctDNA detected in CSF samples at baseline (22.2 months vs 37.6 months, HR = 2.565, *P* = 0.003) (Fig. 2C). The clinical characteristics between subgroups are shown in Additional file 11: Table S1. Baseline CSF ctDNA positivity was associated with larger intracranial tumor size. In multivariate analyses, the detection of ctDNA in baseline CSF samples remained a significant prognostic factor for poor outcomes (HR = 2.683, *P* = 0.006) (Additional file 12: Table S2).

### CSF ctDNA displays unique genetic profiles of metastatic brain tumors

**Fig. 3:**
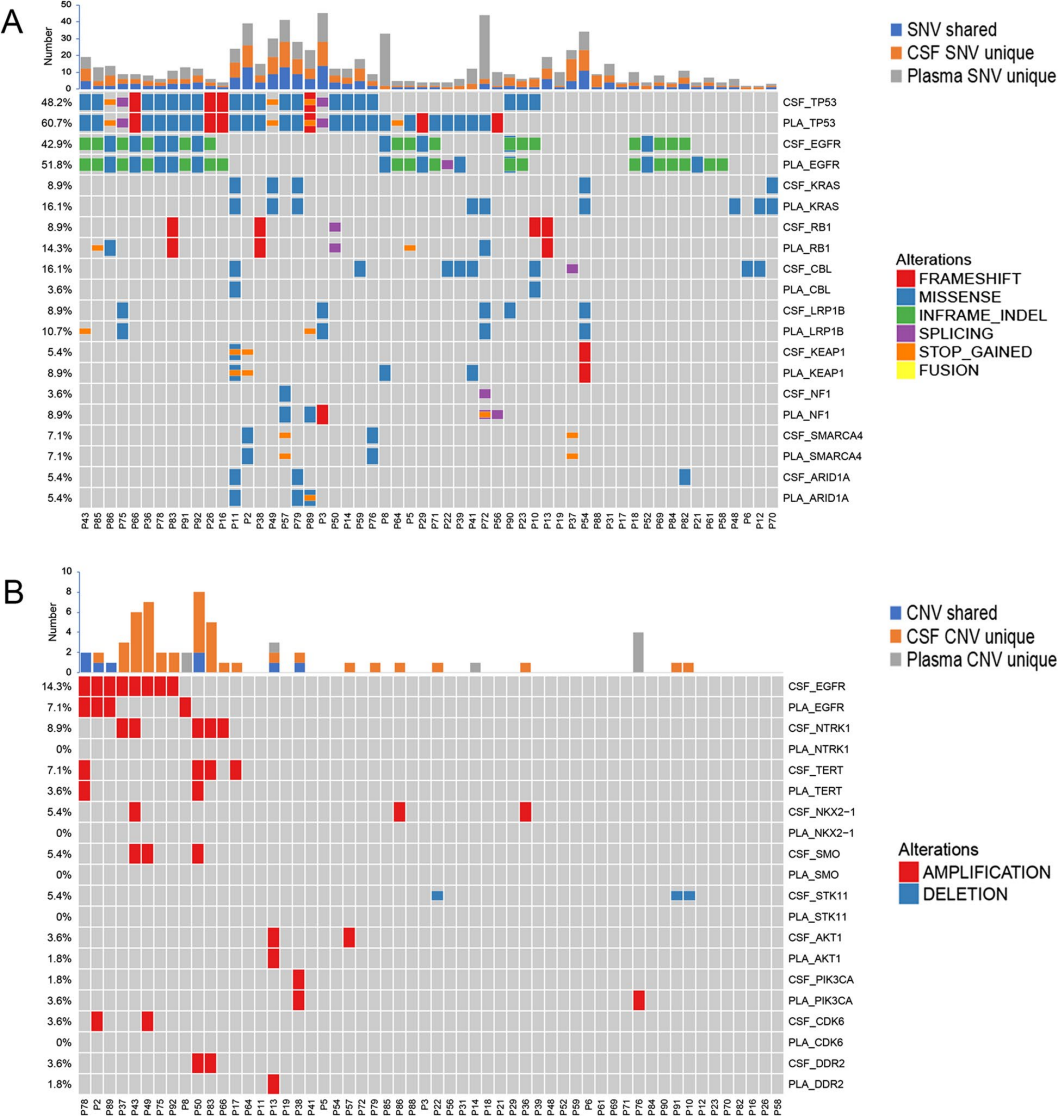
Comparison of genomic profiles between paired CSF and plasma samples. Somatic mutations (**A**) and CNVs (**B**) detected in paired baseline CSF and plasma samples are illustrated by OncoPrint plots. Abbreviations: CSF, cerebrospinal fluid; PLA, plasma; CNV, copy number variation

Among 56 patients with genomic alterations detected in both paired baseline plasma and CSF samples, we compared the genetic profiles between the two sample types (Fig. 3). An average of 56.5% (range: 0–100%) of genomic alterations in plasma were detected in CSF ctDNA, and an average of 83.3% (range: 0–100%) of alterations in driver genes [22] in plasma were detected in CSF ctDNA (Additional file 3: Fig. S3). *EGFR*, *TP53*, and *KRAS* were the most frequently altered genes. The *EGFR* mutation rate was 42.9% (24/56) in CSF ctDNA and 51.8% (29/56) in plasma ctDNA, with a concordance rate of 87.5% between the CSF and plasma samples (Cohen’s kappa coefficient = 0.751). The mutation rate of *TP53* was 48.2% (27/56) in CSF ctDNA and 60.7% (34/56) in plasma, with a concordance rate of 76.8% (Cohen’s kappa coefficient = 0.539). *KRAS* G12C was the predominant alteration in *KRAS* and demonstrated a concordance rate of 83.1% (Cohen’s kappa coefficient = 0.677) between CSF and plasma samples (Additional file 4: Fig. S4).

CNVs were more frequently detected in CSF (39.3%, 22/56) than in plasma (21.4%, 12/56, *P* = 0.04), and the majority of CNVs were unique to CSF samples. *EGFR* copy number gain was the most frequent CNV and was identified in 14.3% (8/56) of CSF and 7.1% (4/56) of plasma samples. Copy number loss of *STK11*, as well as the copy number gain of multiple genes, including *NTRK1*, *NKX2-1*, *SMO*, and *CDK6* were identified exclusively in CSF ctDNA (Fig. 3B). Thus, those data revealed the heterogeneous genetic profiles between CSF and plasma samples with a good concordance in driver mutations.

### Dynamic changes in CSF ctDNA better predict the intracranial tumor response than plasma

**Fig. 4:**
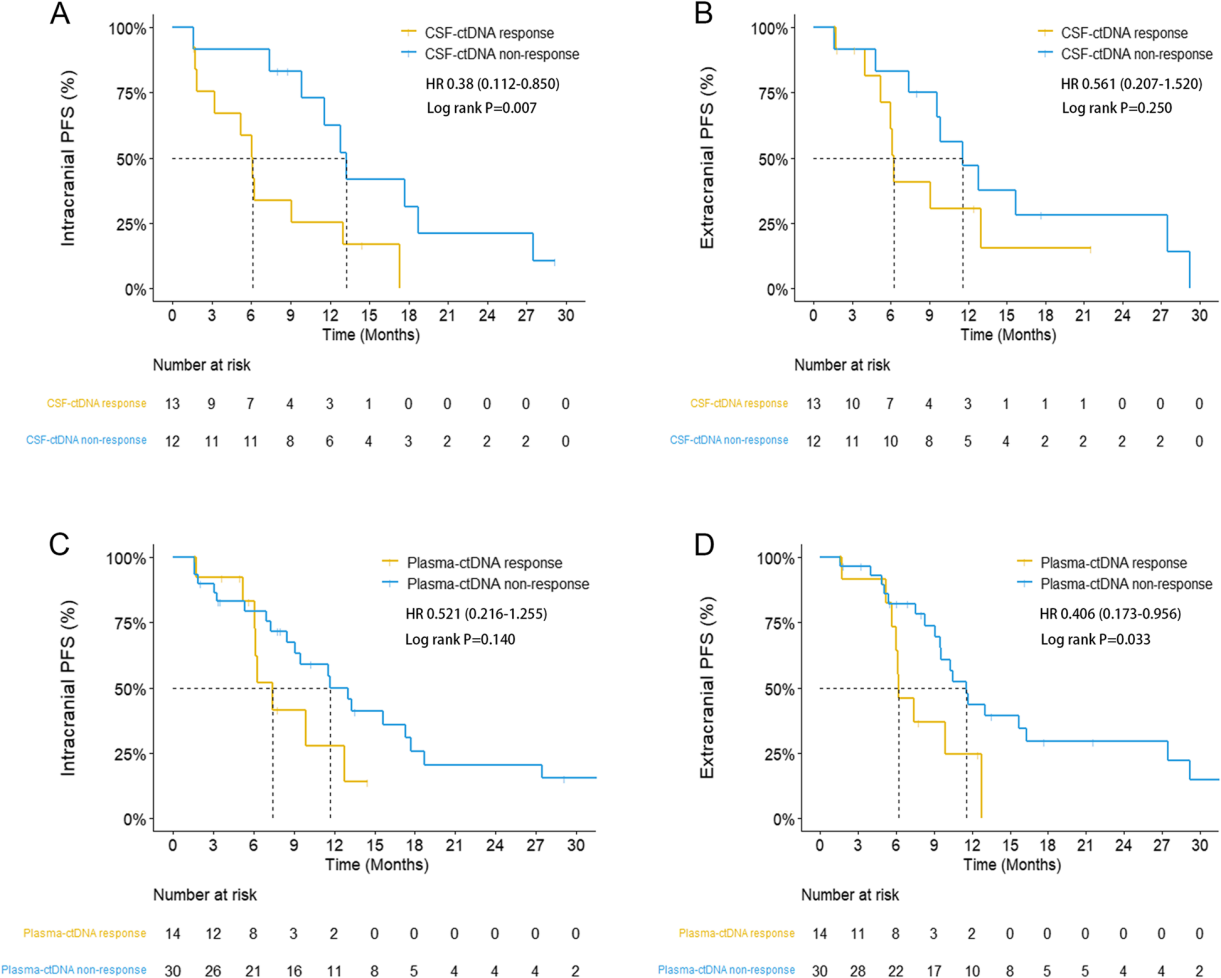
Dynamic changes in CSF ctDNA concentration better predict intracranial tumor response than plasma. Kaplan-Meier curves of intracranial progression-free survival (**A**) and extracranial progression-free survival (**B**) between patients with and without CSF ctDNA response. Kaplan-Meier curves of intracranial progression-free survival (**C**) and extracranial progression-free survival (**D**) between patients with and without plasma ctDNA response. *P*-values were calculated using the log-rank test. Abbreviations: CSF, cerebrospinal fluid; ctDNA, circulating tumor DNA; HR, hazards ratio; PFS, progression-free survival

To investigate whether dynamic changes in CSF ctDNA could reflect intracranial tumor responses and predict clinical outcomes, we collected paired CSF and plasma samples 8 weeks after the initiation of first-line treatment in 51 patients. A “ctDNA response” was defined as a 50% decrease in ctDNA concentration from baseline [24, 25]. Of 25 patients with CSF ctDNA detected at baseline, 12 patients had a CSF ctDNA response, and 13 had no CSF ctDNA response after 8 weeks of treatment. The clinical characteristics and treatment regimens were comparable between the groups (Additional file 11: Table S3). The median intracranial progression-free survival (PFS) of patients with CSF ctDNA response was significantly longer than that of patients without CSF ctDNA response (13.27 months vs 6.13 months, HR = 0.308, *P* = 0.017) (Fig. 4A). The extracranial PFS of patients with CSF ctDNA response was longer than that of patients without CSF ctDNA response (11.57 months vs 6.20 months); however, the difference was not significant (*P* = 0.250) (Fig. 4B).

We further assessed the concordance between the radiographic tumor response and dynamic changes in CSF-ctDNA concentrations. We found a concordance rate of 76.0% between the CSF ctDNA response and radiographic intracranial tumor response evaluated by enhanced brain magnetic resonance imaging examinations (Cohen’s kappa coefficient = 0.522, *P* = 0.008) (Additional file 5: Fig. S5 and Additional file 6: Fig. S6). However, the concordance rate was only 50.0% between the plasma ctDNA response and radiographic intracranial tumor response (Cohen’s kappa coefficient = −0.34, *P* = 0.813). In multivariate analyses, CSF ctDNA response remained a favorable predictive factor for intracranial PFS, regardless of treatment regimens (Additional file 11: Table S4).

Of 44 patients with ctDNA-positive plasma at baseline, 30 patients had plasma ctDNA response after 8 weeks of treatment, and 14 patients had no plasma ctDNA response after 8 weeks, with similar clinical characteristics between the two subsets (Additional file 11: Table S5). Patients with plasma ctDNA response had significantly longer extracranial PFS than patients without plasma ctDNA response (11.57 months vs 6.20 months, HR = 0.406, *P* = 0.033) (Fig. 4D). A trend of longer intracranial PFS was observed in patients with plasma ctDNA response than those without plasma ctDNA response (11.70 months vs 7.37 months, *P* = 0.140); however, the finding was not statistically significant (Fig. 4C). A concordance rate of 72.7% between the plasma ctDNA response and radiographic extracranial tumor response was also observed (Cohen’s kappa coefficient = 0.394, *P* = 0.009) (Additional file 5: Fig. S5 and Additional file 6: Fig. S6).

**Fig. 5:**
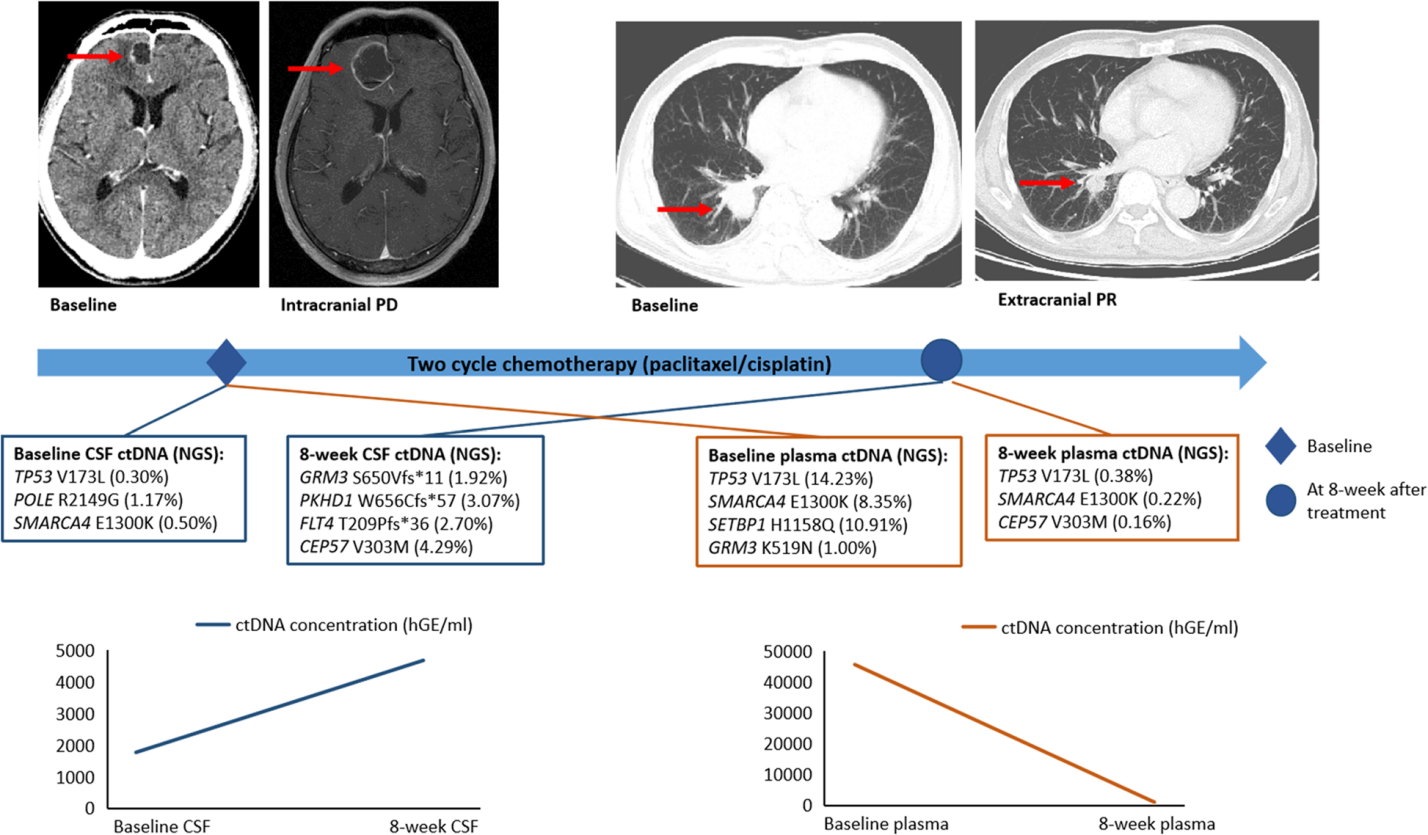
A representative case where the changes in ctDNA concentration of CSF and plasma samples reflected the responses of intracranial and extracranial tumors, respectively. P76 had unique genetic alterations in baseline CSF and plasma samples, and received paclitaxel combined with cisplatin as the first-line treatment. At 8 weeks after two cycles of treatment, a partial response was achieved in the thoracic lesion according to CT scans, while disease progressed in intracranial lesions according to brain MRIs. CSF ctDNA concentrations increased and plasma ctDNA concentrations decreased respectively. Abbreviations: CSF, cerebrospinal fluid; NGS, next-generation sequencing; PD, progression disease; PR, partial response

Figure 5 shows a representative case where the changes of ctDNA in CSF and plasma correlated with the intracranial and extracranial responses, respectively. Patient 76 (P76) had unique genetic alterations in baseline CSF and plasma samples, and received two cycles of paclitaxel combined with cisplatin as the first-line treatment. Eight weeks after the initiation of treatment, a partial response was achieved in the thoracic lesion (as shown on the CT scan), while the intracranial lesion progressed, as revealed by brain MRI. Notably, the ctDNA concentration increased in CSF, whereas it decreased in plasma.

### Evolution of baseline clonal and subclonal mutations in CSF ctDNA during systematic treatments

**Fig. 6:**
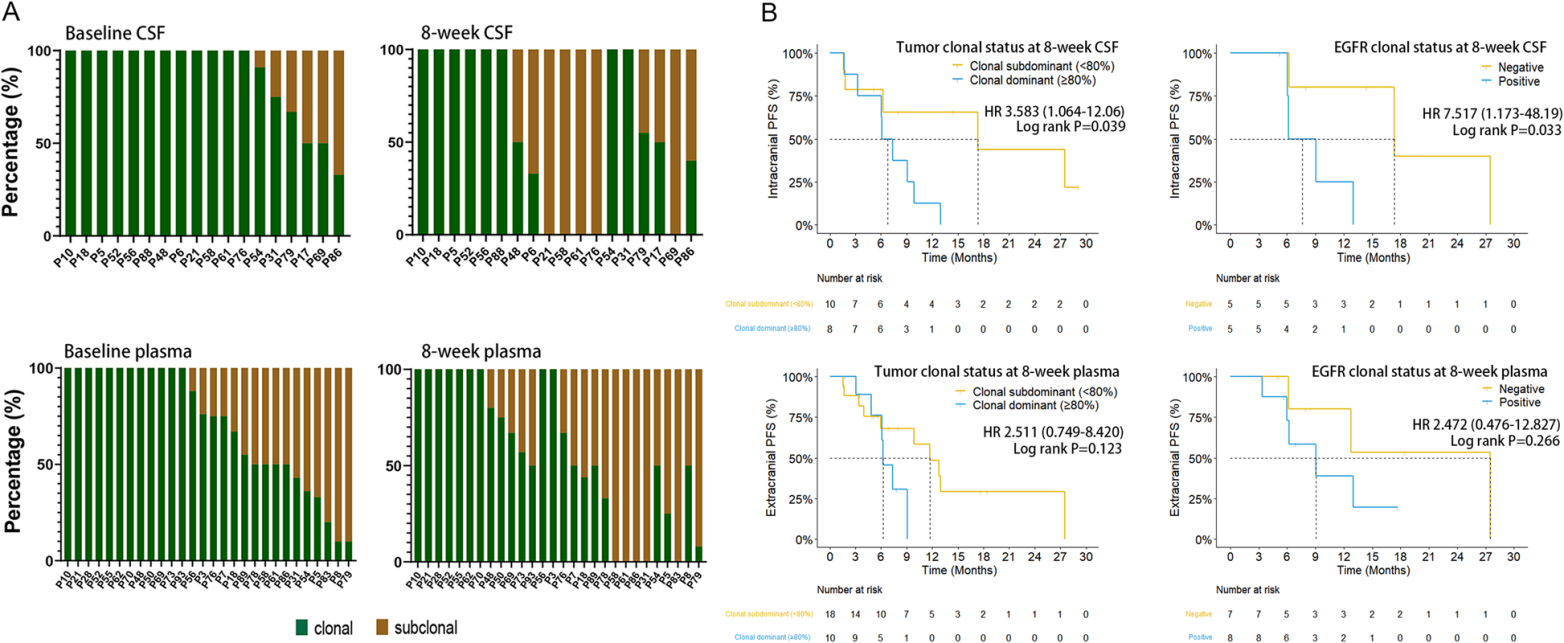
Evolution of clonal and subclonal mutations in CSF and plasma after treatment. **A** Percentage of clonal and subclonal mutations at baseline and 8 weeks after treatment in CSF and plasma. The definition of clonal and subclonal mutations is based on their baseline VAFs (clonal: VAF ≥ 25% * maxVAF in baseline samples) and any acquired mutations after treatment are classified into subclonal subgroups. **B** Kaplan-Meier curves of intracranial and extracranial progression-free survival according to clonal mutations and *EGFR* clones 8 weeks after treatment. *P*-values were calculated using the log-rank test. Abbreviations: CSF, cerebrospinal fluid; HR, hazards ratio; PFS, progression-free survival

To investigate the evolution of baseline clonal and subclonal mutations, and their association with treatment responses, we first defined clonal and subclonal mutations based on their VAF and the max VAF in the baseline samples, as described in the Methods section. At baseline, at least one subclonal mutation was identified in 30% (6/18) of CSF samples, and in 57.1% (16/28) of plasma samples (Fig. 6A). *EGFR* was enriched as the clonal mutation in CSF and plasma samples (Additional file 7: Fig. S7). The newly-acquired mutations after 8 weeks of treatment that were not detectable in baseline samples were grouped as subclonal mutations, regardless of their VAFs. As shown in Fig. 6A, the overall proportion of clonal mutations in post-treatment CSF samples primarily decreased due to the clearance of clonal mutations and the acquisition of new subclonal mutations. (Additional file 8: Fig. S8 and Additional file 9: Fig. S9).

Patients whose clonal mutations accounted for over 80% of mutations detected in the CSF samples that were collected 8 weeks after treatment had shorter intracranial PFS (6.13 months vs 17.33 months, HR = 3.785, *P* = 0.039). However, the maintenance of clonal mutations in post-treatment plasma samples was not a significant prognostic factor for extracranial (Fig. 6B) and intracranial (Additional file 10: Fig. S10) disease; only a trend of shorter PFS was observed for patients with over 80% of baseline-defined clonal mutations in 8-week plasma samples. We then focused on patients who received EGFR-TKIs and found that the detection of baseline-defined clonal *EGFR* mutations in the paired CSF samples 8 weeks after treatment was a poor predictive factor for intracranial PFS (HR = 7.76, *P* = 0.033, Fig. 6B), while the differences in the extracranial PFS were not statistically significant (Additional file 10: Fig. S10). A trend of shorter extracranial PFS was also observed in patients with clonal *EGFR* mutations in plasma 8 weeks after treatment compared to patients negative for clonal *EGFR* mutations (9.07 months vs 27.50 months, HR = 2.472, *P* = 0.266; Fig. 6B and Additional file 10: Fig. S10).

## Discussion

To the best of our knowledge, this is the largest prospective study investigating the genetic landscape of CSF ctDNA vs plasma ctDNA in NSCLC patients with brain metastases. This study is also the first to demonstrate that dynamic changes in CSF ctDNA could better predict intracranial tumor response than plasma, and can be used to monitor intracranial mutational evolution during treatment.

The presence of the blood-brain barrier prevents the release of ctDNA from intracranial tumors to peripheral plasma, and thus, CSF was considered a promising surrogate to represent the genetic landscape of intracranial lesions. In this study, the ctDNA-positivity rate was 63.7% in baseline CSF samples, which was also consistent with previous studies [26, 27], and may be related to the tumor burden and the distance of brain lesion to its nearest ventricle. More importantly, we found that ctDNA-positivity in baseline CSF samples was an adverse prognostic factor for NSCLC patients with brain metastases, even after adjusting for gene type and other characteristics. Alexandra et al. also reported that ctDNA-positivity in baseline CSF samples was related to high disease burden and poor outcomes in glioma patients [28]. Our results indicated that the presence of ctDNA in baseline CSF may be an early indicator of outcomes in NSCLC patients with brain metastases.

Patients with brain metastases harbored different genetic alterations in brain tumors vs primary tumors and exhibited different treatment responses with poor clinical outcomes [29]. In our previous study, we revealed that driver mutations in metastatic brain tumors were highly concordant with paired primary lung tumors, while genetic alterations in cell cycle regulator genes and the PI3K pathway genes were enriched in brain metastases [30]. Due to the invasiveness of obtaining brain tumor samples, we hypothesized that paired CSF and plasma samples could be used to reflect the genetic profiles of brain metastatic tumors and primary tumors, respectively. In this study, we found a high degree of genetic divergence between the CSF and paired plasma samples, while concordance in driver mutations was observed. A higher number of unique CNVs was also identified in CSF ctDNA than in plasma. A previous study also found that CNVs were enriched in the CSF from leptomeningeal metastases in NSCLC patients [31], thus, indicating that CNVs may contribute to brain metastases in lung cancer patients. The identification of genetic alterations in brain metastases using CSF could facilitate precise treatments for such patients.

One of the advantages of liquid biopsies is that they can facilitate real-time tracking of tumor evolution and the prediction of treatment responses. Dynamic changes in the ctDNA in plasma have been reported to be related to tumor response [32–34]. However, the genetic profiles of plasma ctDNA poorly represent those of brain tumors, and thus, the use of CSF to assess intracranial responses should be explored.

Our study was the first prospective study to reveal that the dynamic changes in CSF ctDNA after treatment could better predict intracranial tumor response than plasma. Patients with CSF ctDNA response 8 weeks after treatments had significantly longer intracranial PFS than patients without CSF ctDNA response. Dynamic changes in CSF ctDNA had better concordance with radiographic intracranial responses than plasma. Notably, when patients had opposing intracranial and extracranial tumor responses, the changes in CSF and plasma ctDNA followed the same trends as the intracranial and extracranial tumor burden, respectively. We then tracked the mutational evolution of CSF and plasma during treatment, and found that CSF harbored more subclonal mutations after treatment than plasma, thus, indicating different evolutionary patterns. The clonal mutations that remained in more than 80% in CSF after 8 weeks also predicted a shorter intracranial PFS. Collectively, our study revealed that dynamic monitoring of CSF ctDNA could be used to predict intracranial tumor response and track the evolution of brain tumors.

This study also had some limitations. First, we could not compare the genetic landscapes between CSF and brain tumor tissues due to the invasiveness of obtaining brain lesion samples. Thus, the unique somatic mutations and CNVs detected in CSF ctDNA might reflect the distinct genomic profiles of metastatic brain tumors. However, we are also aware of the possibility that alterations only detected in CSF may not be detected in plasma samples due to high background noise and varying ctDNA fractions. Second, due to the relatively low level of ctDNA-positivity in CSF, the sample size of patients with serial CSF sampling was small. Finally, though patients meeting the inclusion criteria were enrolled consecutively, we restricted our cohort to NSCLC patients with stable brain metastases. Thus, further exploration using large cohorts of patients with advanced CNS involvement should be warranted in the future.

## Conclusions

In conclusion, our study supported the use of CSF as a liquid biopsy for brain tumors, and investigated the unique genetic profiles of metastatic brain tumors by sequencing CSF ctDNA. CSF ctDNA-positivity at baseline may be an early indicator of adverse outcomes in NSCLC patients with brain metastases. More importantly, dynamic changes in CSF ctDNA could better predict intracranial tumor response than plasma, and track intracranial clonal evolution during treatment.

## Supplementary Information

**Additional file 1:** **Fig. S1** The comparison of NGS results in paired baseline CSF, plasma, and tumor tissue samples.

**Additional file 2:** **Fig. S2** Consistency of genomic alterations between tumor tissues and liquid biopsies.

**Additional file 3:** **Fig. S3** Consistency of genomic alterations between paired CSF and plasma samples.

**Additional file 4:** **Fig. S4** Favorable concordance of EGFR, TP53, and KRAS mutation detection was observed between paired CSF and plasma samples.

**Additional file 5:** **Fig. S5** Dynamic changes in ctDNA concentration and radiographic tumor responses.

**Additional file 6**: **Fig. S6** Concordance of ctDNA response and radiographic response.

**Additional file 7**: **Fig. S7** Mutational profiles of CSF and plasma samples at baseline.

**Additional file 8:** **Fig. S8** The proportion of clonal and subclonal mutations in CSF and plasma samples at baseline and after 8 weeks of treatment. 

**Additional file 9:** **Fig. S9** Mutational profilesof CSF and plasma samples at baseline and after 8 weeks of treatment.

**Additional file 10:** **Fig. S10** The association between extracranial and intracranial PFS with tumor/EGFR clonal status in CSF and plasma samples after 8 weeks of treatment.

**Additional file 11:** **TableS1.**The comparison of clinical characteristics between CSF-ctDNA negative and positive subgroups at baseline (*N* = 92). **Table S2.** Univariate and multivariate Cox regression analyses of overall survival based on baseline variables (*N* = 92). **Table S3.** The comparison of clinical characteristics in patients with CSF ctDNA response and non-response after treatment (*N* = 25). **Table S4.** Univariate and multivariate Cox regression analyses of intracranial progression free survival based on baseline variables (*N* = 25). **Table S5.**The comparison of clinical characteristics in patients with plasma ctDNA response and non-response after treatment (*N* = 44).

## Data Availability

The datasets used and analyzed during the current study are available from the corresponding author upon reasonable request.

## Abbreviations

CNS: Central nervous system
CNV: Copy number variation
CSF: Cerebrospinal fluid
ctDNA: Circulating tumor DNA
ECOG PS: Eastern Cooperative Oncology Group Performance Status
HR: Hazards ratio
MRI: Magnetic resonance imaging
NSCLC: Non-small cell lung cancer
PFS: Progression-free survival
TKI: Tyrosine kinase inhibitor
VAF: Variant allele frequency

## References

[1] Mamon HJ, Yeap BY, Janne PA (2005). High risk of brain metastases in surgically staged IIIA non-small-cell lung cancer patients treated with surgery, chemotherapy, and radiation. J Clin Oncol.

[2] Preusser M, Winkler F, Valiente M (2018). Recent advances in the biology and treatment of brain metastases of non-small cell lung cancer: summary of a multidisciplinary roundtable discussion. ESMO Open.

[3] Kuukasjarvi T, Karhu R, Tanner M (1997). Genetic heterogeneity and clonal evolution underlying development of asynchronous metastasis in human breast cancer. Cancer Res.

[4] Gerlinger M, Rowan AJ, Horswell S (2012). Intratumor heterogeneity and branched evolution revealed by multiregion sequencing. N Engl J Med.

[5] Diehl F, Schmidt K, Choti MA (2008). Circulating mutant DNA to assess tumor dynamics. Nat Med.

[6] Diaz LA, Williams RT, Wu J (2012). The molecular evolution of acquired resistance to targeted EGFR blockade in colorectal cancers. Nature.

[7] Connolly ID, Li Y, Gephart MH (2016). The "Liquid Biopsy": the role of circulating DNA and RNA in central nervous system tumors. Curr Neurol Neurosci Rep.

[8] Doherty CM, Forbes RB (2014). Diagnostic lumbar puncture. Ulster Med J.

[9] De Mattos-Arruda L, Mayor R, Ng CKY (2015). Cerebrospinal fluid-derived circulating tumour DNA better represents the genomic alterations of brain tumours than plasma. Nat Commun.

[10] Shu Y, Wu X, Tong X (2017). Circulating tumor DNA mutation profiling by targeted next generation sequencing provides guidance for personalized treatments in multiple cancer types. Sci Rep.

[11] Jiang BY, Li YS, Guo WB (2017). Detection of driver and resistance mutations in leptomeningeal metastases of NSCLC by next-generation sequencing of cerebrospinal fluid circulating tumor cells. Clin Cancer Res.

[12] Xia H, Xue X, Ding H (2020). Evidence of NTRK1 fusion as resistance mechanism to EGFR TKI in EGFR+ NSCLC: results from a large-scale survey of NTRK1 fusions in chinese patients with lung cancer. Clin Lung Cancer.

[13] Li M, Hou X, Zheng L (2022). Utilizing phenotypic characteristics of metastatic brain tumors to improve the probability of detecting circulating tumor DNA from cerebrospinal fluid in non-small-cell lung cancer patients: development and validation of a prediction model in a prospective cohort study. ESMO Open.

[14] Bolger AM, Lohse M, Usadel B (2014). Trimmomatic: a flexible trimmer for Illumina sequence data. Bioinformatics.

[15] Koboldt DC, Zhang Q, Larson DE (2012). VarScan 2: somatic mutation and copy number alteration discovery in cancer by exome sequencing. Genome Res.

[16] Newman AM, Bratman SV, Stehr H (2014). FACTERA: a practical method for the discovery of genomic rearrangements at breakpoint resolution. Bioinformatics.

[17] Song Z, Cai Z, Yan J (2019). Liquid biopsies using pleural effusion-derived exosomal DNA in advanced lung adenocarcinoma. Transl Lung Cancer Res.

[18] Newman AM, Bratman SV, To J (2014). An ultrasensitive method for quantitating circulating tumor DNA with broad patient coverage. Nat Med.

[19] Chaudhuri AA, Chabon JJ, Lovejoy AF (2017). Early detection of molecular residual disease in localized lung cancer by circulating tumor DNA profiling. Cancer Discov.

[20] Mao X, Zhang Z, Zheng X (2017). Capture-based targeted ultradeep sequencing in paired tissue and plasma samples demonstrates differential subclonal ctDNA-releasing capability in advanced lung cancer. J Thorac Oncol.

[21] Strickler JH, Loree JM, Ahronian LG (2018). Genomic landscape of cell-free DNA in patients with colorectal Cancer. Cancer Discov.

[22] Mosele F, Remon J, Mateo J (2020). Recommendations for the use of next-generation sequencing (NGS) for patients with metastatic cancers: a report from the ESMO precision medicine working group. Ann Oncol.

[23] Chakravarty D, Gao J, Phillips SM, et al: OncoKB: A Precision Oncology Knowledge Base. JCO Precis Oncol 2017, 2017 10.1200/PO.17.00011 PMC5586540 28890946

[24] Goldberg SB, Narayan A, Kole AJ (2018). Early assessment of lung cancer immunotherapy response via circulating tumor DNA. Clin Cancer Res.

[25] Nabet BY, Esfahani MS, Moding EJ (2020). Noninvasive early identification of therapeutic benefit from immune checkpoint inhibition. Cell.

[26] Pentsova EI, Shah RH, Tang J (2016). Evaluating cancer of the central nervous system through next-generation sequencing of cerebrospinal fluid. J Clin Oncol.

[27] Wang Y, Springer S, Zhang M (2015). Detection of tumor-derived DNA in cerebrospinal fluid of patients with primary tumors of the brain and spinal cord. Proc Natl Acad Sci U S A.

[28] Miller AM, Shah RH, Pentsova EI (2019). Tracking tumour evolution in glioma through liquid biopsies of cerebrospinal fluid. Nature.

[29] Brastianos PK, Carter SL, Santagata S (2015). Genomic characterization of brain metastases reveals branched evolution and potential therapeutic targets. Cancer Discov.

[30] Wang H, Ou Q, Li D (2019). Genes associated with increased brain metastasis risk in non-small cell lung cancer: Comprehensive genomic profiling of 61 resected brain metastases versus primary non-small cell lung cancer (Guangdong Association Study of Thoracic Oncology 1036). Cancer.

[31] Li YS, Jiang BY, Yang JJ (2018). Unique genetic profiles from cerebrospinal fluid cell-free DNA in leptomeningeal metastases of EGFR-mutant non-small-cell lung cancer: a new medium of liquid biopsy. Ann Oncol.

[32] Cabel L, Riva F, Servois V (2017). Circulating tumor DNA changes for early monitoring of anti-PD1 immunotherapy: a proof-of-concept study. Ann Oncol.

[33] Song Y, Hu C, Xie Z (2020). Circulating tumor DNA clearance predicts prognosis across treatment regimen in a large real-world longitudinally monitored advanced non-small cell lung cancer cohort. Transl Lung Cancer Res.

[34] Wang Z, Cheng Y, An T (2018). Detection of EGFR mutations in plasma circulating tumour DNA as a selection criterion for first-line gefitinib treatment in patients with advanced lung adenocarcinoma (BENEFIT): a phase 2, single-arm, multicentre clinical trial. Lancet Respir Med.

