# Correction: Dynamic monitoring of cerebrospinal fluid circulating tumor DNA to identify unique genetic profiles of brain metastatic tumors and better predict intracranial tumor responses in non-small cell lung cancer patients with brain metastases: a prospective cohort study (GASTO 1028)

**DOI:** 10.1186/s12916-022-02718-1

**Published:** 2023-01-08

**Authors:** Meichen Li, Jing Chen, Baishen Zhang, Juan Yu, Na Wang, Delan Li, Yang Shao, Dongqin Zhu, Chuqiao Liang, Yutong Ma, Qiuxiang Ou, Xue Hou, Likun Chen

**Affiliations:** 1 Department of Medical OncologyState Key Laboratory of Oncology in South ChinaCollaborative Innovation Center for Cancer Medicine, Sun Yat-Sen University Cancer Center, Guangzhou, China; 2 Department of Oncology, The First Affiliated Hospital of Jinan University, Guangzhou, China; 3 Chemotherapy Department 2, Zhongshan City People’s Hospital, Zhongshan, China; 4 School of Public Health, Nanjing Medical University, Nanjing, China; 5 Nanjing Geneseeq Technology Inc, Nanjing, China

**Correction: BMC Med 20, 398 (2022)**

**https://doi.org/10.1186/s12916-022-02595-8**

The original article [1] contained two errors in Fig. 4:

1. The labels of the two groups – response vs. non-response – were mistakenly switched.
2. The HR in panel A was incorrectly stated as 0.38 instead of 0.308.

The figure has been updated in the original article to reflect these amendments.
