# Supplementary figures and images for: Dynamic monitoring of cerebrospinal fluid circulating tumor DNA to identify unique genetic profiles of brain metastatic tumors and better predict intracranial tumor responses in non-small cell lung cancer patients with brain metastases: a prospective cohort study (GASTO 1028)
Source: BMC Med. 2022 Nov 14;20:398. doi: 10.1186/s12916-022-02595-8 (PMC9661744; doi:10.1186/s12916-022-02595-8)

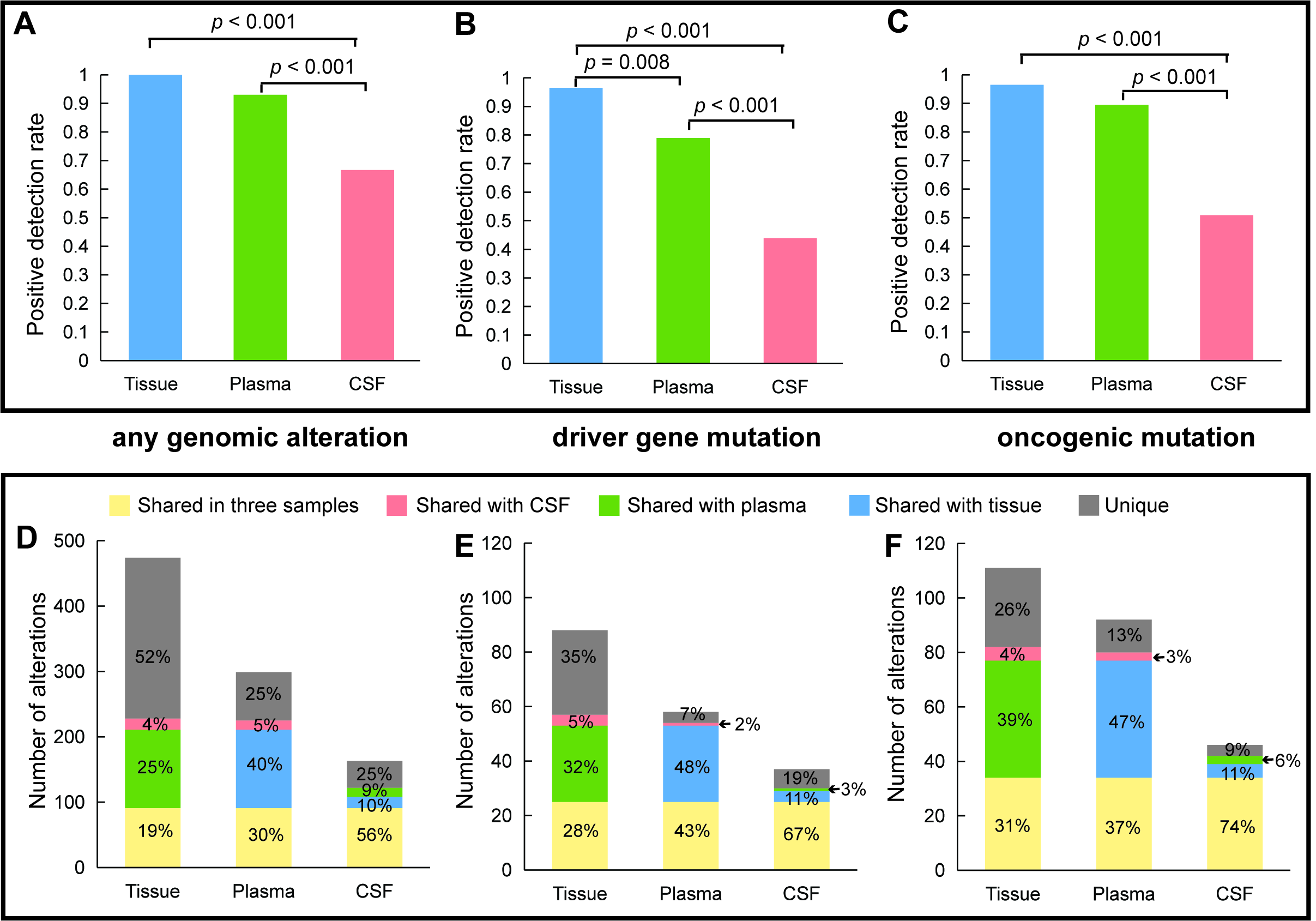

Supplement: Supplementary file 1 — Additional file 1: Fig. S1 The comparison of NGS results in paired baseline CSF, plasma, and tumor tissue samples. [file 12916_2022_2595_MOESM1_ESM.tif]

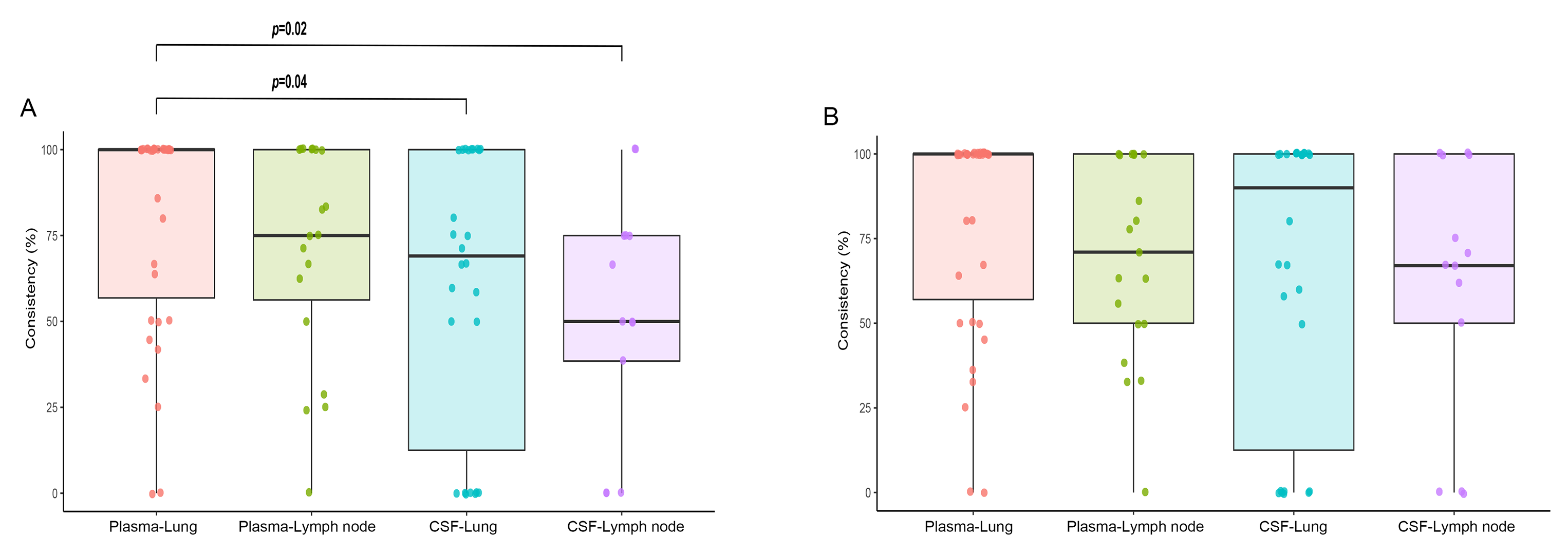

Supplement: Supplementary file 2 — Additional file 2: Fig. S2 Consistency of genomic alterations between tumor tissues and liquid biopsies. [file 12916_2022_2595_MOESM2_ESM.tif]

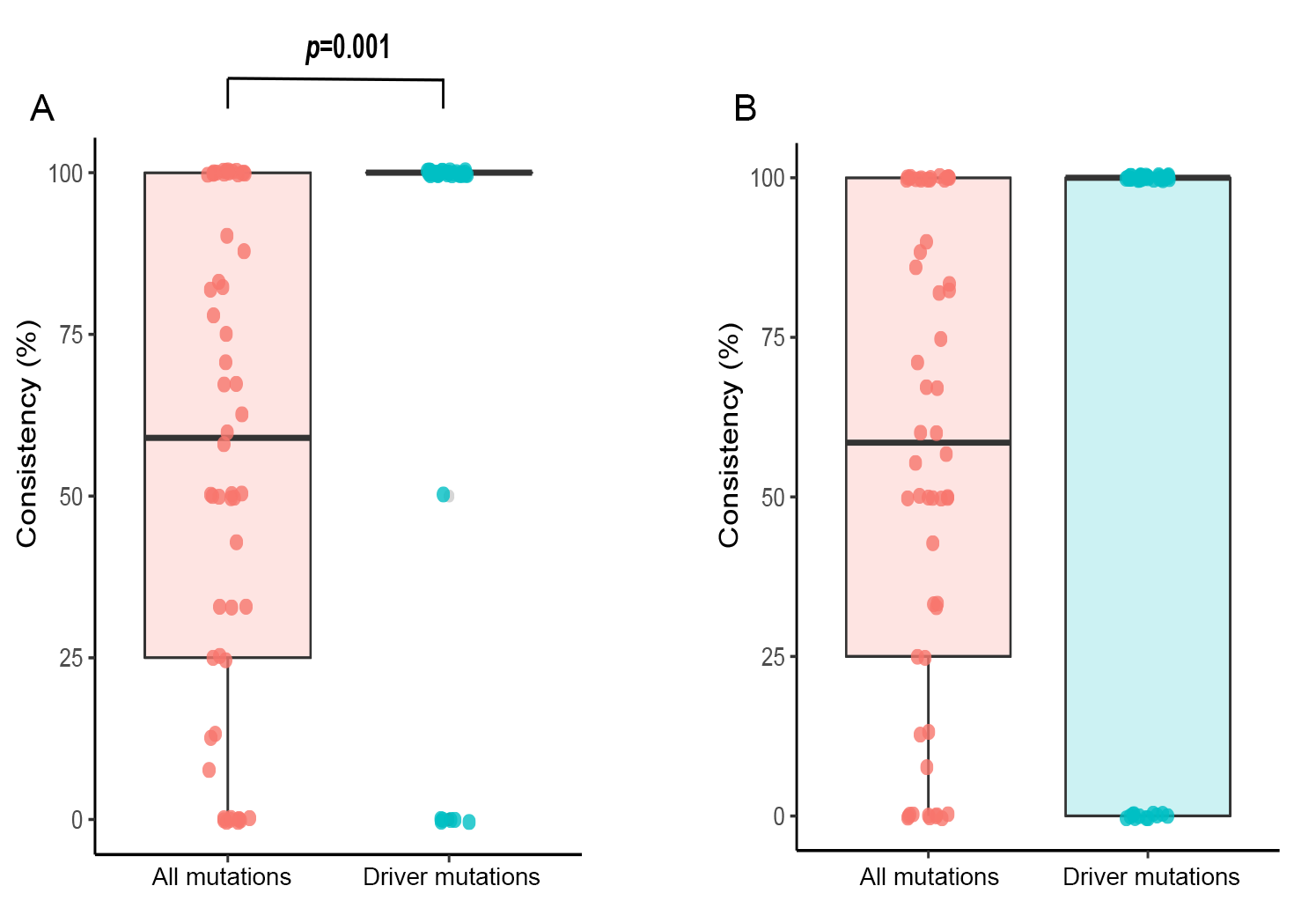

Supplement: Supplementary file 3 — Additional file 3: Fig. S3 Consistency of genomic alterations between paired CSF and plasma samples. [file 12916_2022_2595_MOESM3_ESM.tif]

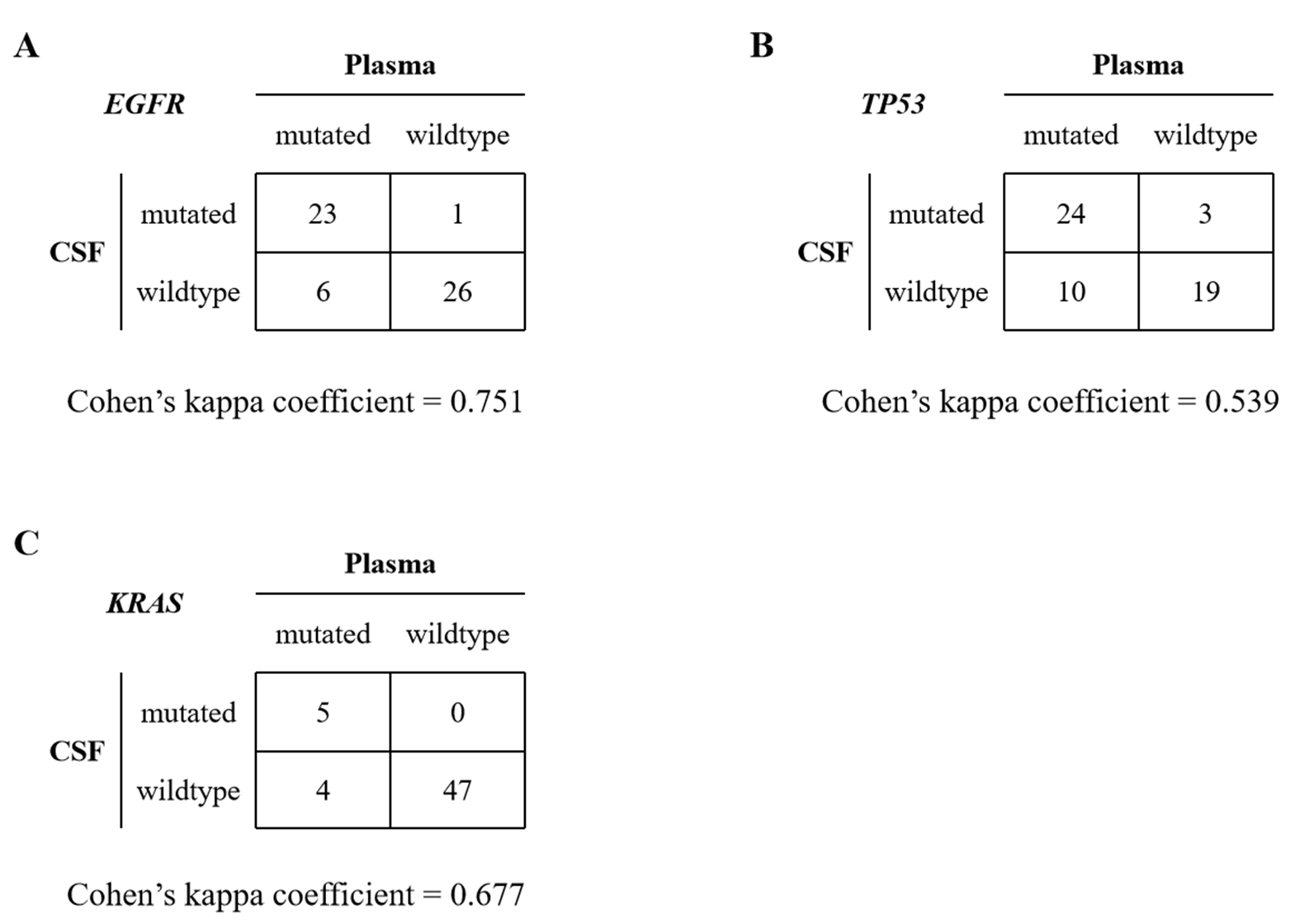

Supplement: Supplementary file 4 — Additional file 4: Fig. S4 Favorable concordance of EGFR, TP53, and KRAS mutation detection was observed between paired CSF and plasma samples. [file 12916_2022_2595_MOESM4_ESM.tif]

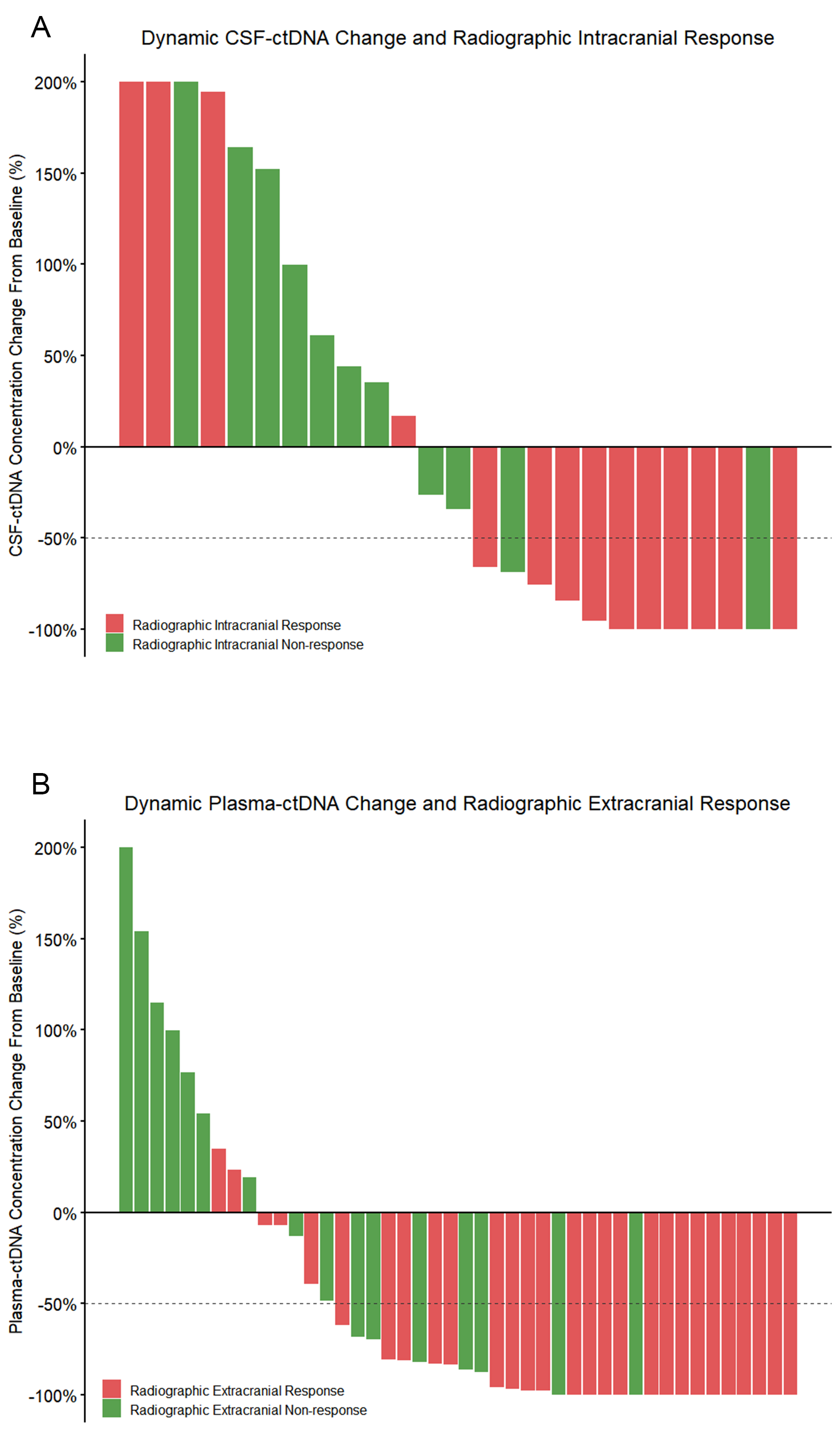

Supplement: Supplementary file 5 — Additional file 5: Fig. S5 Dynamic changes in ctDNA concentration and radiographic tumor responses. [file 12916_2022_2595_MOESM5_ESM.tif]

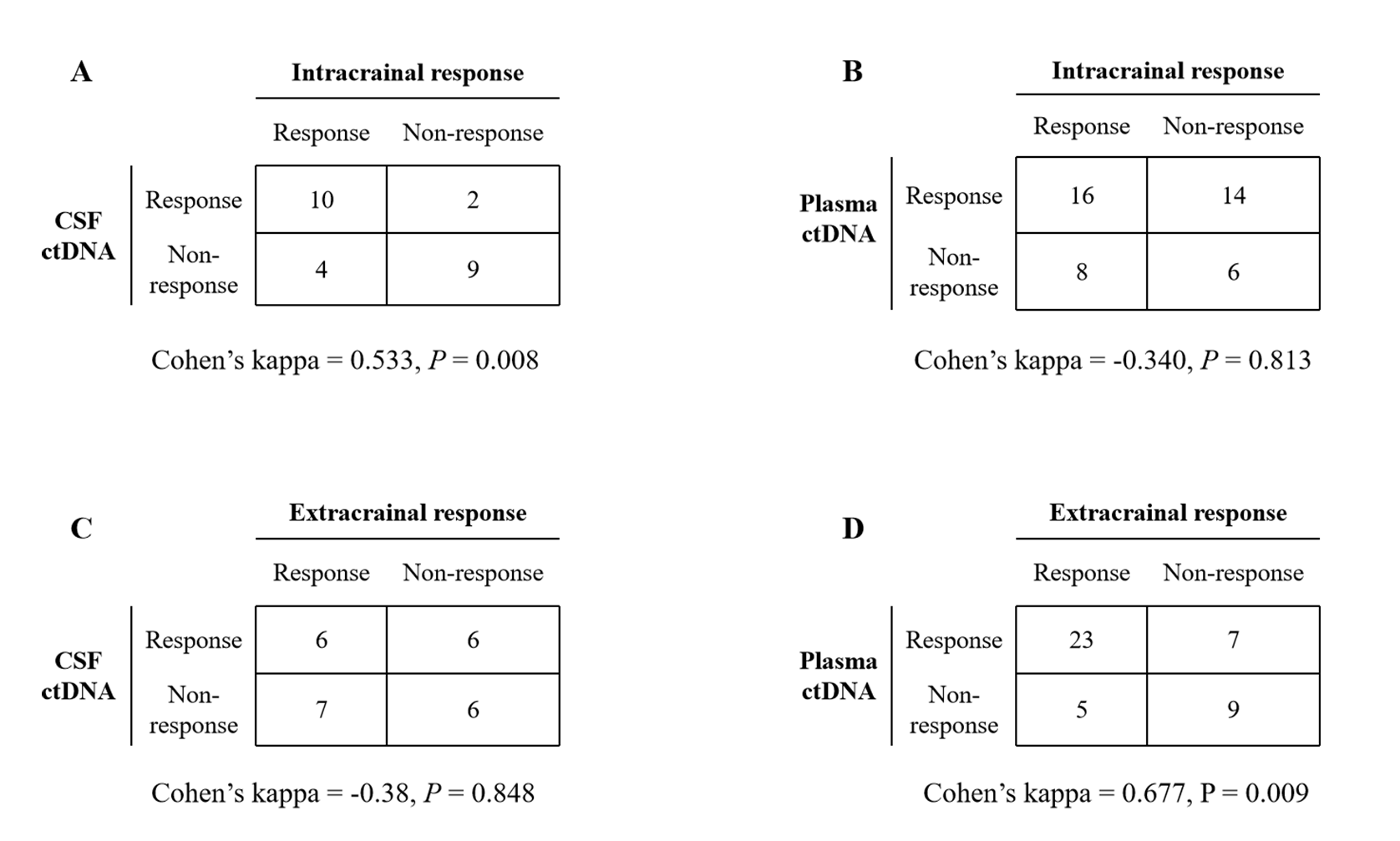

Supplement: Supplementary file 6 — Additional file 6: Fig. S6 Concordance of ctDNA response and radiographic response. [file 12916_2022_2595_MOESM6_ESM.tif]

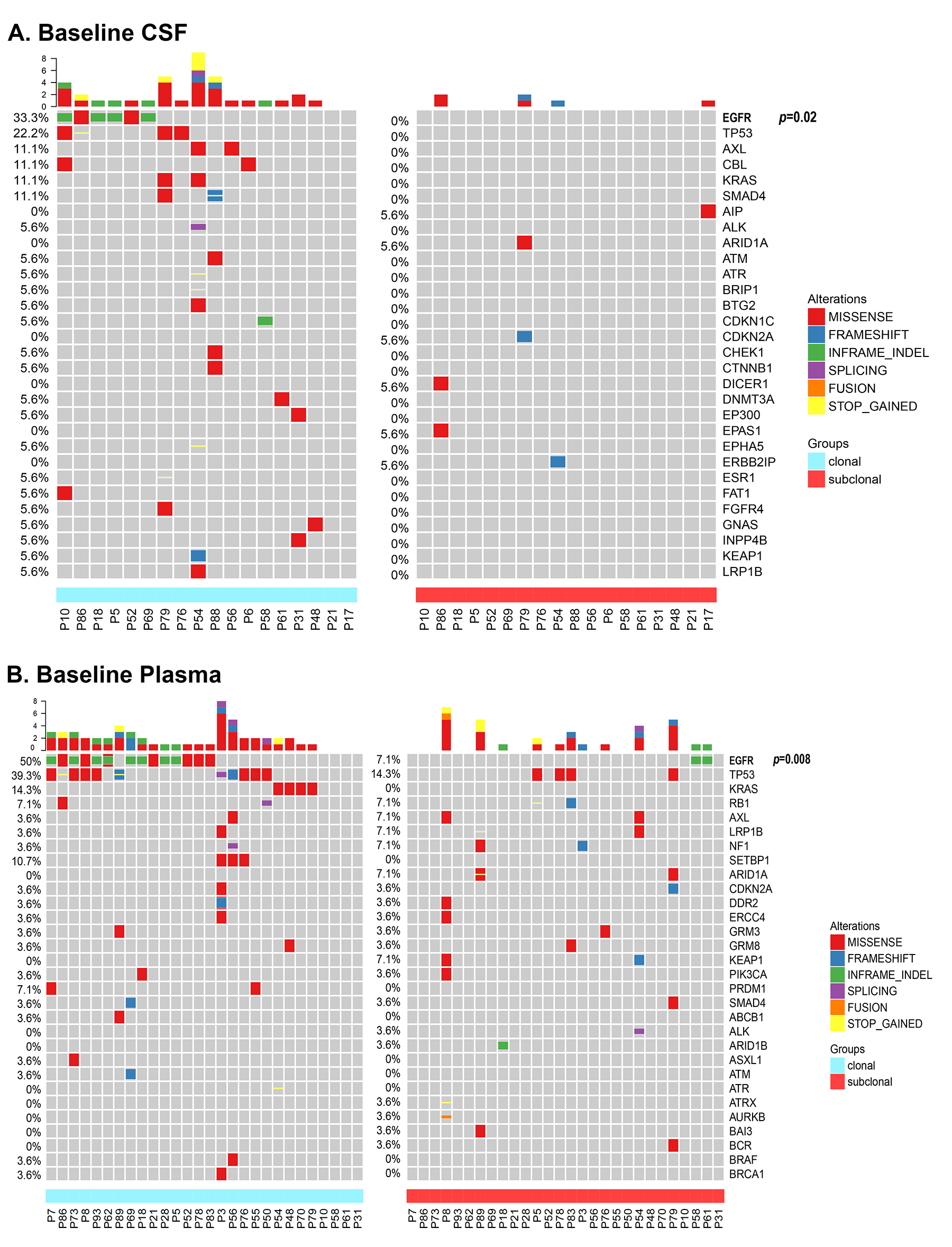

Supplement: Supplementary file 7 — Additional file 7: Fig. S7 Mutational profiles of CSF and plasma samples at baseline. [file 12916_2022_2595_MOESM7_ESM.tif]

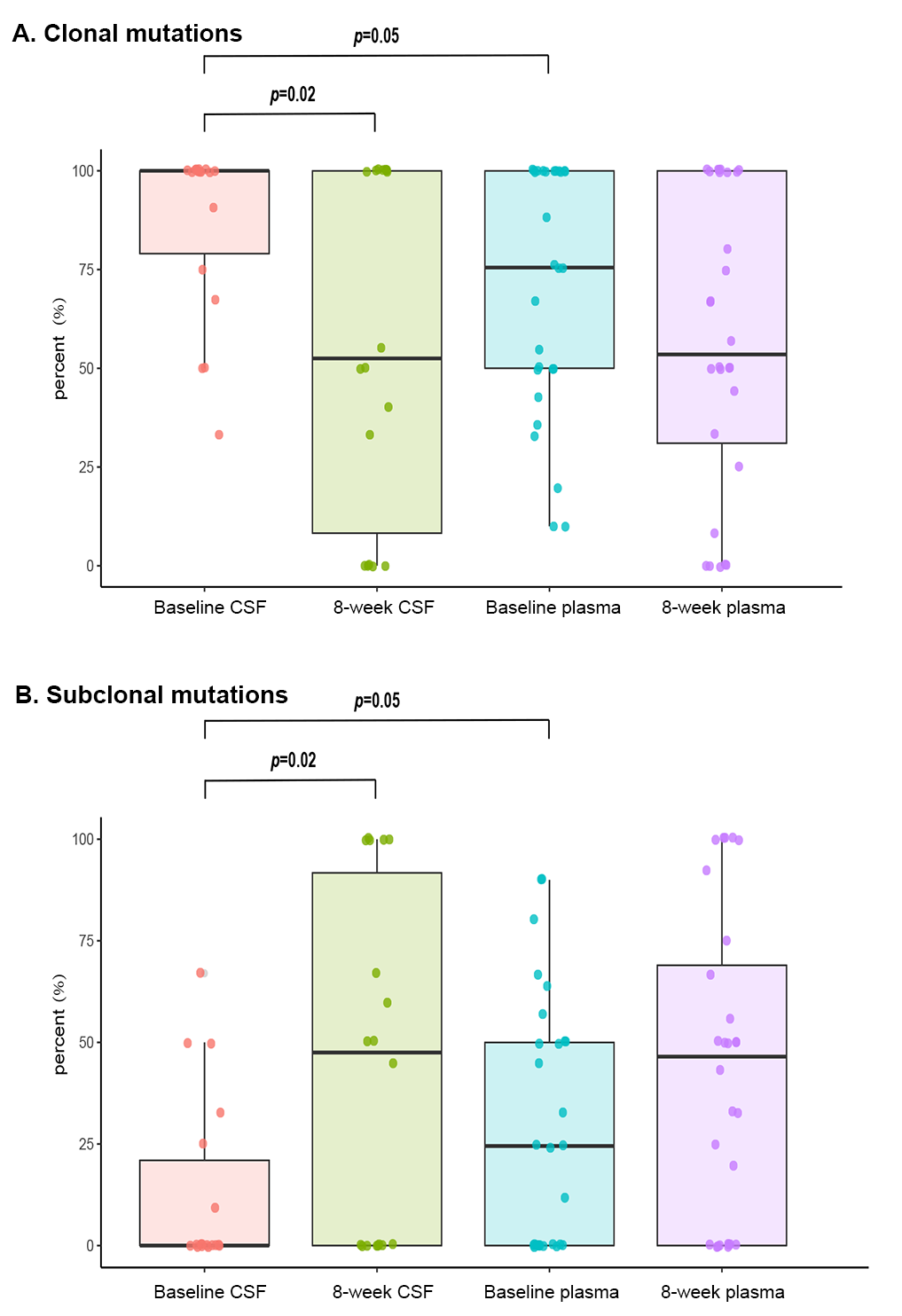

Supplement: Supplementary file 8 — Additional file 8: Fig. S8 The proportion of clonal and subclonal mutations in CSF and plasma samples at baseline and after 8 weeks of treatment. [file 12916_2022_2595_MOESM8_ESM.tif]

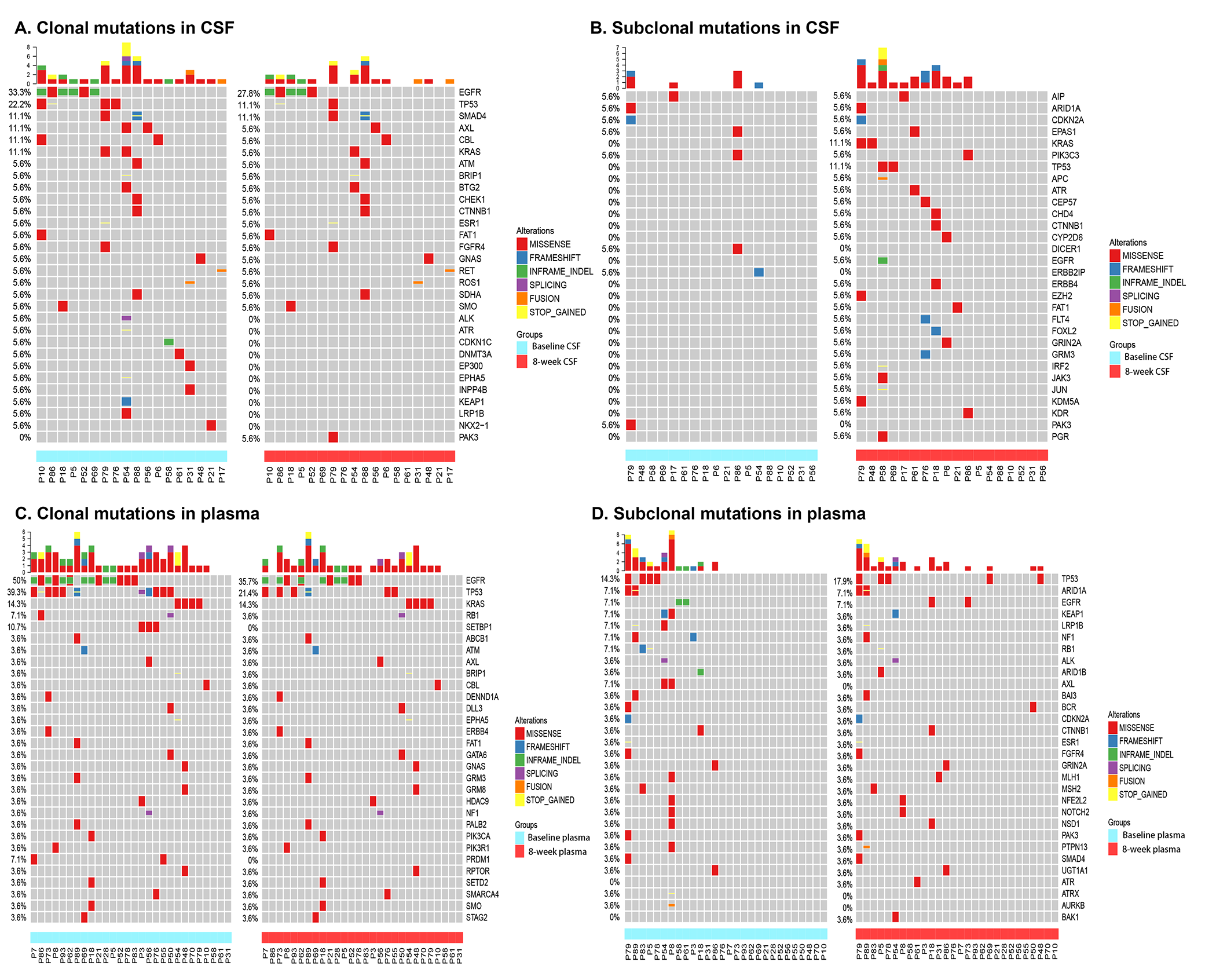

Supplement: Supplementary file 9 — Additional file 9: Fig. S9 Mutational profilesof CSF and plasma samples at baseline and after 8 weeks of treatment. [file 12916_2022_2595_MOESM9_ESM.tif]

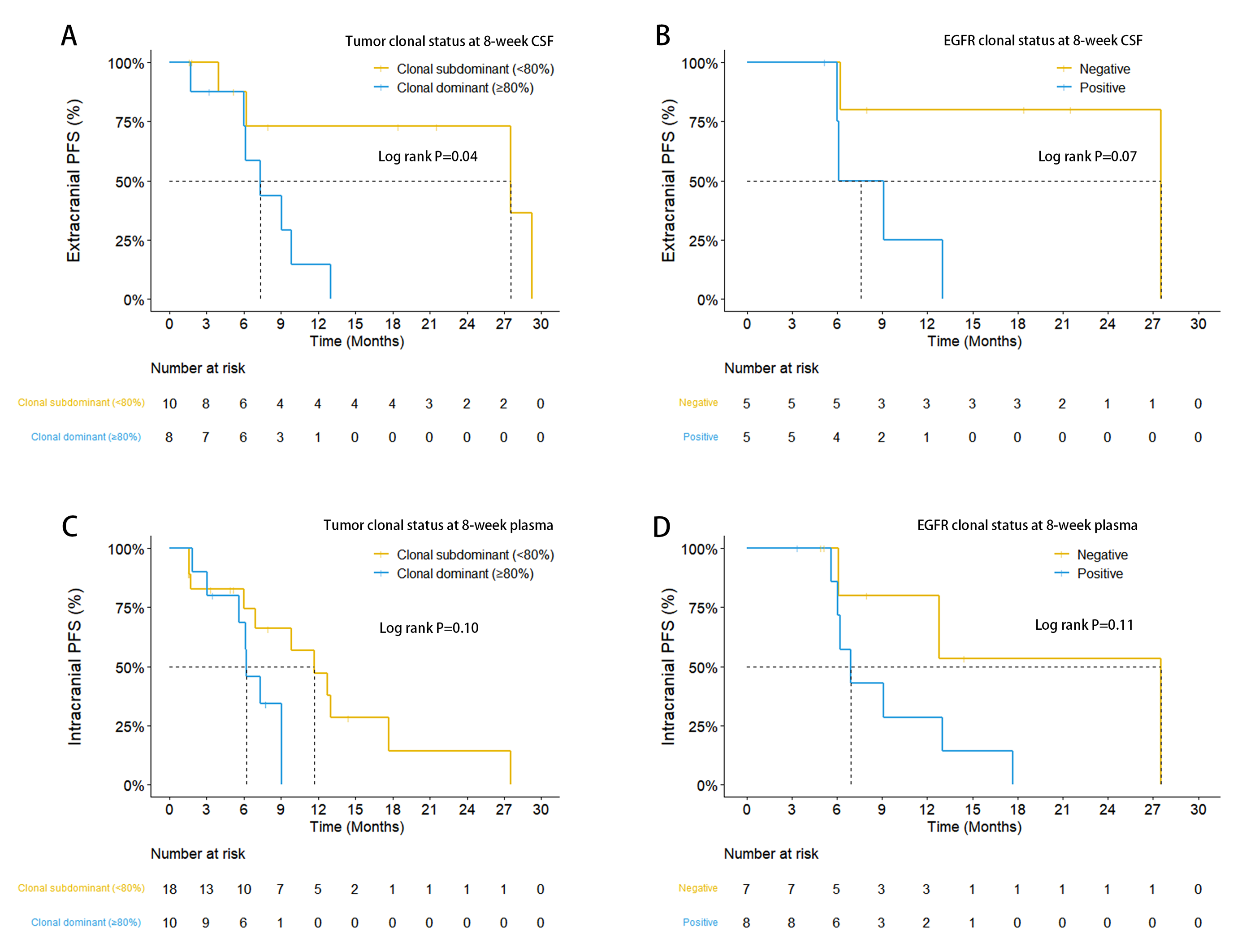

Supplement: Supplementary file 10 — Additional file 10: Fig. S10 The association between extracranial and intracranial PFS with tumor/EGFR clonal status in CSF and plasma samples after 8 weeks of treatment. [file 12916_2022_2595_MOESM10_ESM.tif]
