# Supplementary material for: Dynamic monitoring of cerebrospinal fluid circulating tumor DNA to identify unique genetic profiles of brain metastatic tumors and better predict intracranial tumor responses in non-small cell lung cancer patients with brain metastases: a prospective cohort study (GASTO 1028)
Source: BMC Med. 2022 Nov 14;20:398. doi: 10.1186/s12916-022-02595-8 (PMC9661744; doi:10.1186/s12916-022-02595-8)
Supplement: Supplementary file 11 — Additional file 11: TableS1.The comparison of clinical characteristics between CSF-ctDNA negative and positive subgroups at baseline (N = 92). Table S2. Univariate and multivariate Cox regression analyses of overall survival based on baseline variables (N = 92). Table S3. The comparison of clinical characteristics in patients with CSF ctDNA response and non-response after treatment (N = 25). Table S4. Univariate and multivariate Cox regression analyses of intracranial progression free survival based on baseline variables (N = 25). Table S5.The comparison of clinical characteristics in patients with plasma ctDNA response and non-response after treatment (N = 44). [file 12916_2022_2595_MOESM11_ESM.docx]

**Table S1. The comparison of clinical characteristics between CSF-ctDNA negative and positive subgroups at baseline (N = 92).**

| **Characteristics** | **CSF-ctDNA negative**  **No. (%)** | **CSF-ctDNA positive**  **No. (%)** | ***P* value ^c^** |
| --- | --- | --- | --- |
| **No. of patients** | 34 | 58 | - |
| **Age,** median(range) | 57.5 (37-74) | 57.5 (31-72) | 0.608 |
| **Sex**  Male  Female | 22 (64.7)  12 (35.3) | 37 (63.8)  21 (36.2) | 0.930 |
| **Histology type**  Adenocarcinoma  Non-adenocarcinoma | 29 (85.3)  5 (14.7) | 49 (84.5)  9 (15.5) | 0.917 |
| **Smoking status**  Smokers  Nonsmokers | 15 (44.1)  19 (55.9) | 26 (44.8)  32 (55.2) | 0.947 |
| **ECOG PS**  0-1  ≥ 2 | 28 (82.4)  6 (17.6) | 35 (60.3)  23 (39.7) | 0.028 |
| **CNS symptoms**  Yes  No | 15 (44.1)  19 (55.9) | 34 (58.6)  24 (41.4) | 0.178 |
| **Brain tumor number**  1-3  ≥ 4 | 15 (44.1)  19 (55.9) | 16 (27.6)  42 (72.4) | 0.105 |
| **Maximum brain tumor size (mm)**  **Median, (range)** | 14.5 (5-43) | 21.0 (5-47.4) | 0.002 |
| **Thoracic stage ^a^**   1. II   IIIA  IIIB  IIIC | 6 (17.6)  11 (32.4)  16 (47.1)  1 (2.9) | 6 (10.3)  18 (31.0)  24 (41.4)  10 (17.3) | 0.194 |
| **Distant metastases**  Liver  Bone  Adrenal gland | 2 (5.9)  16 (47.1)  4 (11.8) | 9 (15.5)  29 (50.0)  15 (25.9) | 0.203  0.785  0.107 |
| **Gene mutation**  *EGFR* mutation  *ALK* rearrangement  *EGFR/ALK* wild-type | 16 (47.1)  3 (8.8)  15 (44.1) | 28 (48.3)  0 (0.0)  30 (51.7) | 0.068 |
| **Lung-molGPA ^b^**  0-1  1.5-2  2.5-3  3.5-4.5 | 1 (3.0)  7 (20.6)  18 (52.9)  8 (23.5) | 7 (12.1)  16 (27.6)  26 (44.8)  9 (15.5) | 0.319 |
| **First-line treatment**  Chemotherapy  TKIs  Immunotherapy | 11 (32.3)  19 (55.9)  4 (11.8) | 24 (41.4)  28 (48.3)  6 (10.3) | 0.690 |

Abbreviations: ECOG PS, Eastern Cooperative Oncology Group performance status; CNS, central nervous system; TKIs, Tyrosine kinase inhibitors.

^a^ Thoracic stage was calculated according to 8th American Joint Committee on Cancer (AJCC) staging system.

^b^ An update of the diagnosis-specific Graded Prognostic Assessment (DS-GPA) using molecular markers.

^c^ *P* Values calculated using χ2 or Fisher’s exact test.

**Table S2. Univariate and multivariate Cox regression analyses of overall survival based on baseline variables (N = 92).**

| **variables** | **Univariate analysis** | | **Multivariate analysis** | |
| --- | --- | --- | --- | --- |
|  | **HR (95% CI)** | ***P* value** | **HR (95% CI)** | ***P* value** |
| **Age,**  < 60 vs ≥ 60 | 1.090(0.621-1.912) | 0.763 | - | - |
| **Sex,**  Male vs female | 1.643(0.894-3.022) | 0.110 | - | - |
| **Histology,**  LUAD vs others | 0.788(0.380-1.636) | 0.523 | - | - |
| **Smoking status,**  Smokers vs nonsmokers | 1.757(1.006-3.069) | 0.048 | 1.239(0.679-2.259) | 0.485 |
| **ECOG PS,**  ≥ 2 vs 0-1 | 1.502(0.851-2.650) | 0.160 | - | - |
| **CNS symptoms,**  Yes vs no | 0.949(0.544-1.655) | 0.853 | - | - |
| **Brain tumor number,**  1-3 vs ≥ 4 | 0.764(0.424-1.376) | 0.370 | - | - |
| **Maximum brain tumor size (mm),**  < 20 vs ≥ 20 | 0.644(0.368-1.128) | 0.124 | - | - |
| **Thoracic stage**  I-IIIA vs IIIB-IIIC | 0.702(0.395-1.248) | 0.228 | - | - |
| **Distant metastases**  Liver metastases, yes vs no  Bone metastases, yes vs no  Adrenal gland metastases, yes vs no | 2.464(1.208-5.027)  1.099(0.631-1.916)  2.109(1.140-3.903) | 0.013  0.738  0.017 | 1.744(0.825-3.684)  -  1.047(0.526-2.082) | 0.145  -  0.896 |
| **CSF-ctDNA,**  Positive vs negative | 2.565(1.348-4.880) | 0.003 | 2.683(1.325-5.434) | 0.006 |
| **Plasma-ctDNA,**  Positive vs negative | 1.441(0.518-4.012) | 0.484 | - | - |
| ***EGFR/ALK* mutation,**  Mutant vs wild-type | 0.347(0.196-0.615) | <0.001 | 0.381(0.204-0.709) | 0.002 |

Abbreviations: LUAD, lung Adenocarcinoma; ECOG PS, Eastern Cooperative Oncology Group performance status; CNS, central nervous system; CSF, Cerebrospinal Fluid; ctDNA, circulating tumor DNA; HR, hazard ratio; CI, confidence interval.

**Table S3. The comparison of clinical characteristics in patients with CSF ctDNA response and non-response after treatment (N = 25).**

| **Characteristics** | **CSF ctDNA response**  **No. (%)** | **CSF ctDNA non-response**  **No. (%)** | ***P* value ^b^** |
| --- | --- | --- | --- |
| **No. of patients** | 12 | 13 | - |
| **Age,** median (range) | 54.5 (31-69) | 59 (42-68) | 0.419 |
| **Sex**  Male  Female | 8 (66.7)  4 (33.3) | 8 (61.5)  5 (38.5) | 1.000 |
| **Histological type**  Adenocarcinoma  Non-adenocarcinoma | 10 (83.3)  2 (16.7) | 10 (76.9)  3 (23.1) | 1.000 |
| **Smoking status**  Smokers  Nonsmokers | 6 (50.0)  6 (50.0) | 4 (30.8)  9 (69.2) | 0.428 |
| **ECOG PS**  0-1  ≥ 2 | 5 (41.7)  7 (58.3) | 7 (53.8)  6 (46.2) | 0.543 |
| **CNS symptoms**  Yes  No | 7 (58.3)  5 (41.7) | 7 (53.8)  6 (46.2) | 0.821 |
| **Brain tumor number**  1-3  ≥ 4 | 6 (50.0)  6 (50.0) | 3 (23.1)  10 (76.9) | 0.226 |
| **Maximum brain tumor size (mm),**  Median, (range) | 17.0 (6.0-35.0) | 22.5 (5.0-38.0) | 0.231 |
| ***EGFR* mutation**  Wild type  Mutant | 5 (41.7)  7 (58.3) | 5 (38.5)  8 (61.5) | 1.000 |
| **Lung-molGPA ^a^**  0-1  1.5-2  2.5-3  3.5-4.5 | 1 (8.3)  4 (33.3)  5 (41.7)  2 (16.7) | 1 (7.65)  5 (38.5)  6 (46.2)  1 (7.65) | 0.920 |
| **First-line treatment**  Chemotherapy  TKIs  Immunotherapy | 4 (33.3)  7 (55.3)  1 (8.4) | 5 (38.5)  8 (61.5)  0 (0.0) | 0.566 |

Abbreviations: ECOG PS, Eastern Cooperative Oncology Group performance status; CNS, central nervous system; TKIs, Tyrosine kinase inhibitors.

^a^ An update of the diagnosis-specific Graded Prognostic Assessment (DS-GPA) using molecular markers. ^b^ *P* Values calculated using χ2 or Fisher’s exact test.

**Table S4. Univariate and multivariate Cox regression analyses of intracranial progression free survival based on baseline variables (N = 25).**

| **Variables** | **Univariate analysis** | | **Multivariate analysis** | |
| --- | --- | --- | --- | --- |
|  | **HR (95% CI)** | **P value** | **HR (95% CI)** | **P value** |
| **Age,**  < 60 vs ≥ 60 | 1.099(0.435-2.777) | 0.841 | - | - |
| **Sex,**  Male vs Female | 0.856(0.341-2.147) | 0.740 | - | - |
| **Histology,**  LUAD vs Others | 1.433(0.477-4.386) | 0.530 | - | - |
| **Smoking status,**  Smokers vs non-smokers | 0.897(0.354-2.273) | 0.819 | - | - |
| **ECOG PS,**  0-1 vs ≥ 2 | 0.877(0.361-2.130) | 0.773 | - | - |
| **CNS symptoms,**  Yes vs No | 0.921(0.370-2.293) | 0.859 | - | - |
| **Brain tumor number,**  1-3 vs ≥ 4 | 4.505(1.435-14.136) | 0.010 | 5.494(1.626-18.52) | 0.006 |
| **Maximum brain tumor size (mm),**  < 20 vs ≥ 20 | 1.721(0.667-4.443) | 0.262 | - | - |
| **CSF ctDNA,**  response vs  non-response | 0.308(0.112-0.850) | 0.007 | 0.262(0.092-0.749) | 0.013 |
| ***EGFR* mutation,**  Mutant vs wild-type | 0.588(0.228-1.519) | 0.273 | - | - |

Abbreviations: LUAD, lung Adenocarcinoma; ECOG PS, Eastern Cooperative Oncology Group performance status; CNS, central nervous system; CSF, Cerebrospinal Fluid; ctDNA, circulating tumor DNA; HR, hazard ratio; CI, confidence interval.

**Table S5. The comparison of clinical characteristics in patients with plasma ctDNA response and non-response after treatment (N = 44).**

| **Characteristics** | **Plasma ctDNA response**  **No. (%)** | **Plasma ctDNA non-response No. (%)** | ***P* value ^c^** |
| --- | --- | --- | --- |
| **No. of patients** | 30 | 14 | - |
| **Age,** median (range) | 56 (45-74) | 55 (31-69) | 0.195 |
| **Sex**  Male  Female | 17 (56.7)  13 (43.3) | 9 (64.3)  5 (35.7) | 0.632 |
| **Histological type**  Adenocarcinoma  Non-adenocarcinoma | 23 (76.7)  7 (23.3) | 14 (100.0)  0 (0.0) | 0.078 |
| **Smoking status**  Smokers  Nonsmokers | 10 (33.3)  20 (66.7) | 7 (50.0)  7 (50.0) | 0.290 |
| **ECOG PS**  0-1  ≥ 2 | 18 (60.0)  12 (40.0) | 8 (57.1)  6 (42.9) | 0.858 |
| **CNS symptoms**  Yes  No | 14 (46.7)  16 (53.3) | 9 (64.3)  5 (35.7) | 0.276 |
| **Brain tumor number**  1-3  ≥ 4 | 13 (43.3)  17 (56.7) | 4 (28.6)  10 (71.4) | 0.349 |
| **Maximum brain tumor size (mm),**  Median, (range) | 16.2 (6.0-43.0) | 22.5 (5.0-38.0) | 0.164 |
| **Thoracic stage ^a^**  I-II  IIIA  IIIB  IIIC | 3 (10.0)  9 (30.0)  16 (53.3)  2 (6.7) | 0 (0.0)  6 (42.9)  5 (35.7)  3 (21.4) | 0.229 |
| **Distant metastases**  Liver  Bone  Adrenal gland | 4 (13.3)  20 (66.7)  5 (16.7) | 2 (14.3)  7 (50.0)  6 (42.9) | 1.000  0.290  0.132 |
| ***EGFR* mutation**  Wild type  Mutant | 12 (40.0)  18 (60.0) | 6 (42.9)  8 (57.1) | 0.858 |
| **Lung-molGPA ^b^**  0-1  1.5-2  2.5-3  3.5-4.5 | 2 (6.7)  9 (30.0)  14 (46.7)  5 (16.6) | 0 (0.0)  6 (42.9)  6 (42.9)  2 (14.2) | 0.691 |
| **First-line treatment**  Chemotherapy  TKIs  Immunotherapy | 11 (36.7)  18 (60.0)  1 (3.3) | 5 (35.7)  8 (57.2)  1 (7.1) | 0.852 |

Abbreviations: ECOG PS, Eastern Cooperative Oncology Group performance status; CNS, central nervous system; TKIs, Tyrosine kinase inhibitors.

^a^ Thoracic stage was calculated according to 8th American Joint Committee on Cancer (AJCC) staging system.

^b^ An update of the diagnosis-specific Graded Prognostic Assessment (DS-GPA) using molecular

markers.

^c^ *P* Values calculated using χ2 or Fisher’s exact test.
